# Supervised Learning of Protein Melting Temperature: Cross‐Species vs. Species‐Specific Prediction

**DOI:** 10.1002/prot.70019

**Published:** 2025-07-14

**Authors:** Sebastián García López, Jesper Salomon, Wouter Boomsma

**Affiliations:** ^1^ Department of Computer Science—DIKU University of Copenhagen Copenhagen Denmark; ^2^ Enzyme Research Division Novonesis Kongens Lyngby Denmark

**Keywords:** contrastive representation learning, ESM, fine‐tuning, inverse folding, melting temperature prediction, transfer‐learning

## Abstract

Protein melting temperatures are important proxies for stability, and frequently probed in protein engineering campaigns, for instance for enzyme discovery and protein optimization. With the emergence of large datasets of melting temperatures for diverse natural proteins, it has become possible to train models to predict this quantity, and the literature has reported impressive performance values in terms of Spearman rho. The high correlation scores suggest that it should be possible to accurately predict melting temperature changes in engineered variants, and to reliably identify naturally thermostable proteins. However, in practice, results in these settings are often disappointing. In this paper, we explore this apparent discrepancy. We show that Spearman rho over cross‐species data gives an overly optimistic impression of prediction performance, and that this metric reflects the ability to distinguish global differences in amino acid composition between species, rather than the specific effects of genetic variation. We proceed by investigating whether cross‐species training on melting temperature is beneficial at all, compared to training specific models for each species. We address this question using four different transfer‐learning approaches and a fine‐tuning procedure. Surprisingly, we consistently find no benefit of cross‐species training. We conclude that (1) current models for supervised prediction of melting temperature perform substantially worse than the literature suggests, and (2) that reliable transfer across species is still a challenging problem. An implementation of this work is available at https://github.com/deltadedirac/thermocontrast_tm.

## 1 Introduction

Reliable prediction of protein thermal stability is a long‐standing challenge in protein engineering, particularly for enzyme optimization, where it is a strong indicator for functional integrity under thermal stress. Special focus has been on the prediction of the *changes* in stability induced by mutations, also referred to as ΔΔG. Over the last decades, a long list of algorithms have been developed for this purpose, ranging from early models such as FoldX [1] and Rosetta [2, 3, 4], to deep learning approaches based on convolutional and graph neural networks ([5, 6, 7]), and most recently approaches building on large pre‐trained models [8].

The task of predicting *absolute* stabilities (ΔG) is generally considered more difficult, although some recent success in this area has been reported using (unsupervised) inverse‐folding models [9]. Supervised learning in this area has traditionally been challenging due to lack of large scale data, but new high‐throughput experimental studies are beginning to provide sufficiently large, consistent datasets to make this feasible. An example is melting temperature data, which is a useful proxy for absolute stability, with the advantage that it can be measured reproducibly in high‐throughput assays and, unlike other measures of protein stability (e.g., assays for stability under specific conditions), is directly comparable across proteins. This makes it a valuable general optimization target, frequently correlating with stability to complex stress factors [10]. A recent large‐scale dataset of melting temperature of 48 000 proteins across 13 species [11], combined with standardized splits [12], has made the melting temperature prediction problem accessible to the machine learning community. This has led to a flurry of new prediction methods, typically using pre‐trained foundation models as the basis for supervised training. Two common approaches are (1) to train a *new* task‐specific model using static embedding vectors from a pre‐trained model as input, or (2) to *fine‐tune* the foundation model on task‐specific data, often using some parameter efficient technique to avoid overfitting. Several recent studies have reported impressive prediction performance, with Spearman correlation coefficients above 0.7 [12, 13, 14, 15, 16, 17, 18].

Although progress in this area is highly welcome, the high performance values reported in the literature are somewhat at odds with our expectation about the difficulty of this prediction task. As mentioned, we would generally expect absolute stability prediction to be more challenging than relative stability prediction, where we do not see such high correlation coefficients. But more specifically, it conflicts with our own prior experience with melting temperature prediction in the protein engineering setting, where we have typically seen substantially lower performances than those reported. In this paper, we investigate the origin of this apparent discrepancy as a stepping stone toward a better understanding of the status of supervised learning performance in this domain.

The paper is organized as follows: We start by identifying the basis for the performance discrepancy described above—and show that much of the reported Spearman correlation arises as a consequence of the difference in melting temperature between species, rather than an ability to predict melting temperatures for individual variants. We thus confirm that species‐specific melting temperature remains a challenging problem, with current methods displaying RMS errors of about 6°, which are substantial, given the inner‐species variance. Motivated by the large discrepancy between species, we then investigate whether there is any meaningful transfer of information between species, by comparing to models trained on a single species at a time. We conduct this analysis using a collection of different training approaches: (1) A simple transfer learning approach based on an ESM2 [19] embedding, (2) a transfer‐learning approach combining embeddings from sequence (ESM2) and structure‐based (PiFold [20]) foundation models, (3) a transfer‐learning procedure built on contrastive representation learning [21], (4) a transfer‐learning approach that explicitly anchors the results to a proxy for the optimal growth temperature of the species, and (5) a recently proposed standardized fine‐tuning approach of the ESM2 model [22]. Our results demonstrate that across all these training procedures, we consistently see species‐specific models outperforming globally trained models, despite the substantially smaller datasets. We conclude by discussing the implications of these results in terms of best practices for supervised melting temperature prediction.

## 2 Results

**FIGURE 1:**
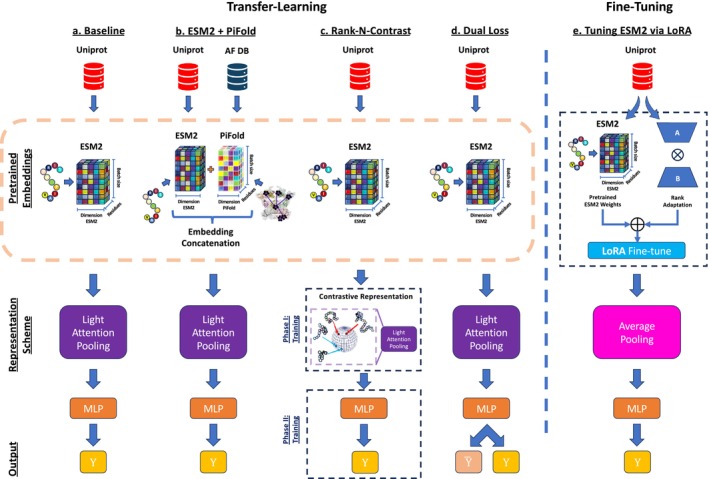
Five different prediction approaches, building on pretrained models. (a) a simple baseline, using ESM2 [19] embeddings pooled using light‐attention; (b) Similar to the baseline, but using a richer featurization consisting of a concatenation of ESM2 and PiFold [20], an inverse‐folding model; (c) an contrastive representation‐learning approach using the Rank‐N‐Contrast procedure; (d) A dual‐loss approach, where melting temperatures are predicted as offsets from the species mean; (e) a LORA‐base fine‐tuning approach.

**FIGURE 2:**
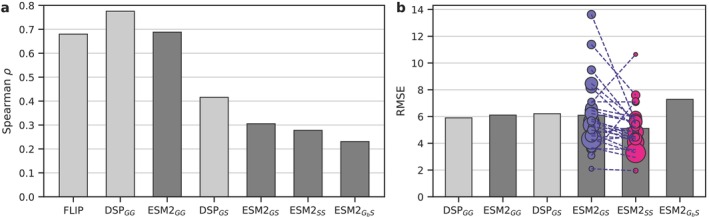
Performance of sequence‐based embeddings to predict melting temperature across species. (a) Performance measured in terms of Spearman rank correlation, (b) Performance measured in terms of RMSE, showing the individual performances per species as circles, scaled by the size of the dataset. The G and S subscripts denote cross‐species (*global*), and *specifies‐specific*, respectively, with the first letter denoting the training scenario and the second denoting the testing scenario. For instance, *GS* denotes a model trained on all data, while being evaluated on each species. $G_{B}$ denotes a model where the dataset was balanced by species during training (see *Methods*). Note how much of the correlation in the second and third bars of the left plot arise simply from the fact that the correlation is measured across all species.

We start by establishing a simple model architecture as a baseline for our subsequent analyses (Figure 1). The model takes ESM2 embeddings [19] as input and produces melting temperature values as output. For simplicity, we use a classic transfer‐learning setup with frozen embeddings (no fine‐tuning). To aggregate the per‐position embeddings into a single output, we use a light attention layer, as this is known to outperform simple averaging over the length of the protein [23, 24]. We analyze our performance on the Meltome Atlas dataset [11], using the standard splits as provided in the FLIP benchmark [12]. Figure 2a (three left‐most bars) shows that this baseline marginally outperforms the results reported in the original FLIP paper, while providing somewhat lower performance than a recently published dedicated melting temperature predictor DeepSTABp [25], demonstrating that our baseline implementation serves as a reasonable representative of the current capabilities in the field. See *Materials and Methods* and Table S1 for details.

### 2.1 Discrepancy Explained: Spearman Rho Is a Poor Metric

**FIGURE 3:**
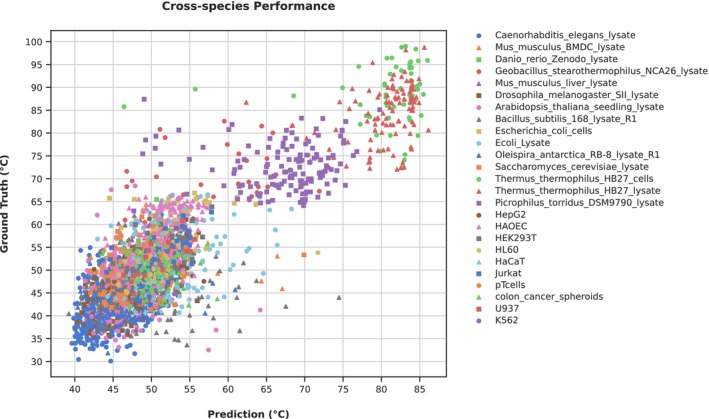
Scatterplot depicting the performance of the baseline cross‐species model. Each point in the scatterplot is encoded by a unique marker and color combination, denoting the species of origin for the corresponding protein. This visualization complements the observations reported in Figure 2, highlighting that high Spearman rho arises as a consequence of cross‐species variation, and that such analyses provide an overly optimistic picture of the prediction performance within species.

The ESM2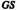 bar in Figure 2a is equivalent to the third (ESM2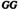), but evaluates the Spearman correlation for each specifies *individually* and reports the average. The dramatic difference of this result compared to the three first bars explains the discrepancy discussed above. Clearly, much of the observed correlation found in the first two bars is due simply to global melting temperature differences between species, rather than the ability to determine melting temperature differences for different proteins within a species. This Figure effect is apparent if we consider a scatter plot of the predictions colored by species (Figure 3). This suggests that basing conclusions solely on the correlation of predictions with the ground truth may lead to misleading conclusions, and highlights the need to include additional metrics in the assessment of model performance. To illustrate, if we instead measure our performance in terms of root mean square error (RMSE), we find no notable discrepancy between the cross‐species and per‐species assessment of our model (Figure 2b).

### 2.2 Cross‐Species vs. Species‐Specific Models

We have established that evaluating performance per species aligns more closely with the objective of interest, namely assessing the impact of variation within species. A natural question is whether there is any cross‐species transfer of information at all, or whether we would obtain better models if we simply trained separate models for each species. The training of cross‐species vs. species‐specific models represents a trade‐off: on one hand, one might expect that cross‐species models could benefit from larger datasets and could provide greater generalization capabilities; on the other hand, a specialized species‐specific model might obtain a better fit with fewer parameters.

Assessing this question empirically with our baseline model on the FLIP dataset, we see that training species‐specific models (ESM2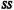, Figure 2b, fifth column) seem to provide improved performance compared the globally trained model, suggesting that training larger cross‐species models is not beneficial in this setting. We see this both in terms of the average RMSE and as a general trend across the different species (Figure 2b, circles). We note that the lack of performance of the global model is not related to the imbalance of data set sizes between species. In fact, rebalancing the databaset during training leads to worse results (ESM2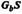). Instead, the problem is likely related to the same behavior that led to the exaggerated performance of the global Spearman correlation: a Simpson‐paradox like issue, where the model focuses on the global trends and disregard the local intra‐species effects (Figure 3).

In order to establish whether this difference between cross‐species models and species‐specific models is a general phenomenon, we probe the effect on a range of different modeling and supervised training procedures. We will introduce them each in turn:

**Approach 0: The baseline** As introduced above, this is a simple transfer learning approach from frozen ESM2 [19] embeddings (Figure 1a).

**Approach 1: Richer embeddings** An obvious candidate for improved performance is the source of the embedding itself. We do not expect substantial differences between the embeddings from different protein language models [26], so we focus here on incorporating structural information by combining the ESM2 sequence embedding with embeddings obtained from the PiFold [20] inverse folding model (Figure 1b).

**Approach 2: Contrastive representation learning** Another appealing approach is *contrastive* learning, where a model is trained to enforce pairwise relationships between samples. We follow a recent approach, rank‐N‐contrast [21], which uses a contrastive procedure to learn task‐specific representations, which are subsequently used for the downstream regression phase (1c). The representation learning phase contrasts samples based on their ranking within the target space of continuous labels. For example, if two samples in a batch during training have the highest similarity in terms of melting temperature, they will be enforced to have the shortest distance in representation space. Since the original publication reported a beneficial role of data augmentation, we add a simple data augmentation step in the form of a small Gaussian perturbation of the embedding input vector (see *Materials and Methods* for details).

**Approach 3: Anchoring to optimal growth temperature** Different organisms have different optimal growth temperatures (OGT), which reflects the ability of proteins and biomolecules to maintain their essential functions and structural integrity in different temperature environments. In an attempt to explicitly model this effect, we introduce a procedure that offsets the predictions relative to the optimal growth temperature (OGT) for the respective species (Figure 5). To simplify matters, we will assume that the optimal growth temperature has a constant offset to the average melting temperature of the proteins for that species, and therefore use the average $T_{m}$ of each species as a proxy for the OGT. The model now has two outputs, predicting the average for the species (shared for all proteins of the species), and a protein specific offset from this offset. The corresponding loss function is:

(1) $${ℒ}_{\text{dual}}=\vartheta_{p}+\vartheta_{s}\;\text{where}\;\vartheta_{p}=\mathrm{MSE}\left(\varphi_{T_{m}},y_{T_{m}}\right)$$

(2) $$\vartheta_{s}=\mathrm{MSE}\left({\tilde{y}}_{\mathrm{OGT}},\mu_{T_{m}}\right)$$

(3) $$\varphi_{T_{m}}={\tilde{y}}_{\mathrm{OGT}}+{\tilde{y}}_{\text{bias}}$$

Here, $\varphi_{T_{m}}$ denotes the predicted $T_{m}$, composed of two components: ${\tilde{y}}_{\mathrm{OGT}}$ and ${\tilde{y}}_{\text{bias}}$, representing the two outputs from the final layer of the MLP $T_{m}$ predictor (see Figure 1 and Section 4.2). The term ${\tilde{y}}_{\mathrm{OGT}}$ estimates the species average for each sample, while ${\tilde{y}}_{\text{bias}}$ indicates the deviation in degrees from this average. Additionally, $y_{T_{m}}$ denotes the ground truth melting temperature, and $\mu_{T_{m}}$ is the average $T_{m}$ per species, serving as the reference OGT per species.

**Approach 4: Parameter efficient Fine‐tuning** As our final approach, we use a recently proposed protocol for parameter efficient fine‐tuning of protein language models [22]. Here, rather than training a downstream model from scratch, the pre‐trained model is instead adapted to improve performance on the task, by adding low‐rank update matrices to the dense layers of the language model [27]. Note that we for this protocol use simple average pooling instead of light attention pooling, in line with the published protocol [22].

**FIGURE 4:**
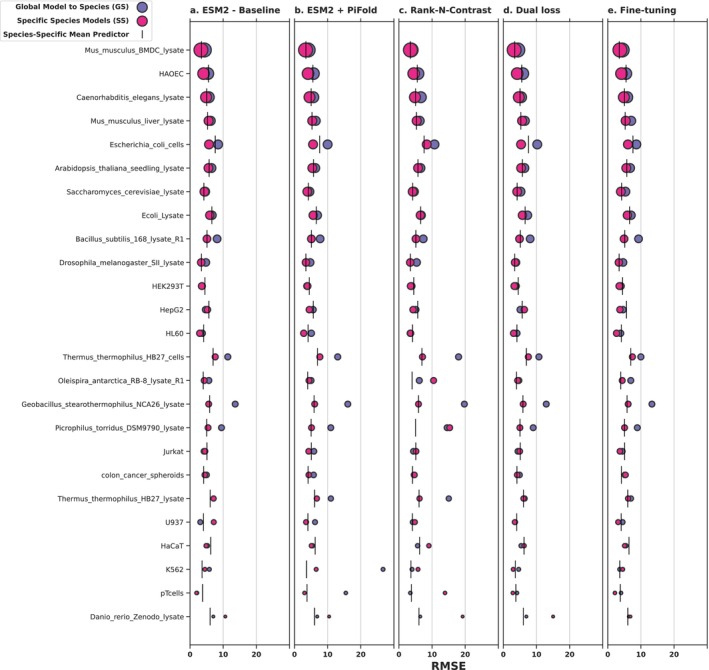
Performance of five methods across species, highlighting the difference between training a single model across species versus training separate models for each species. The size of the circles reflects the size of the datasets for each species. Results from a simple mean‐prediction reference are included for reference.

The results are presented in Figure 4, highlighting the differences across species and across methodological approaches. Overall, the different approaches show remarkably similar behavior, the fine‐tuning approach perhaps showing slightly lower variance on the small datasets, while the rank‐N‐contrast is perhaps slightly less robust. The main take‐away, however, is that the species‐specific models seem to consistently outperform the cross‐species models. We quantify these differences in Table 1, including paired t‐test results that confirm significance of this conclusion in all 5 cases (for results on the individual species, see Tables S1–S6 and Figures S1–S14).

**TABLE 1:** Overview of results in terms of an average root‐mean‐square error (RMSE) and mean relative absolute error (MRAE) for the individual datasets, for models trained on all species at once (cross‐species) or for each species individually (species‐specific).

	Cross‐species		Species‐specific		*t*‐statistic	*p*
	RMSE	MRAE	RMSE	MRAE		
Baseline	6.17 ± 0.11	8.9% (0.1 pp)	4.86 ± 0.09	7.2% (0.1 pp)	11.82	1.34e‐31
ESM2 + PiFold	6.77 ± 0.12	9.3% (0.2 pp)	4.71 ± 0.08	6.9% (0.1 pp)	16.21	8.97e‐57
Rank‐N‐contrast	7.62 ± 0.13	10.4% (0.2 pp)	6.27 ± 0.11	8.4% (0.1 pp)	11.58	2.21e‐30
Dual loss	6.20 ± 0.11	8.7% (0.1 pp)	4.81 ± 0.09	7.0% (0.1 pp)	12.68	5.83e‐36
Fine‐tuning	6.37 ± 0.11	9.2% (0.2 pp)	4.72 ± 0.08	6.9% (0.1 pp)	15.41	1.05e‐51
Mean predictor	11.66 ± 0.15	15.2% (0.2 pp)	5.16 ± 0.06	8.0% (0.1 pp)	28.06	1.77e‐154

*Note*: The compared methods include several transfer learning procedures with static embeddings and a fine‐tuning approach (see main text for details). A simple mean averaging procedure is included as a reference. Row‐wise paired *t*‐tests were performed to assess whether the cross‐species and species‐specific methods were significantly different.

**FIGURE 5:**
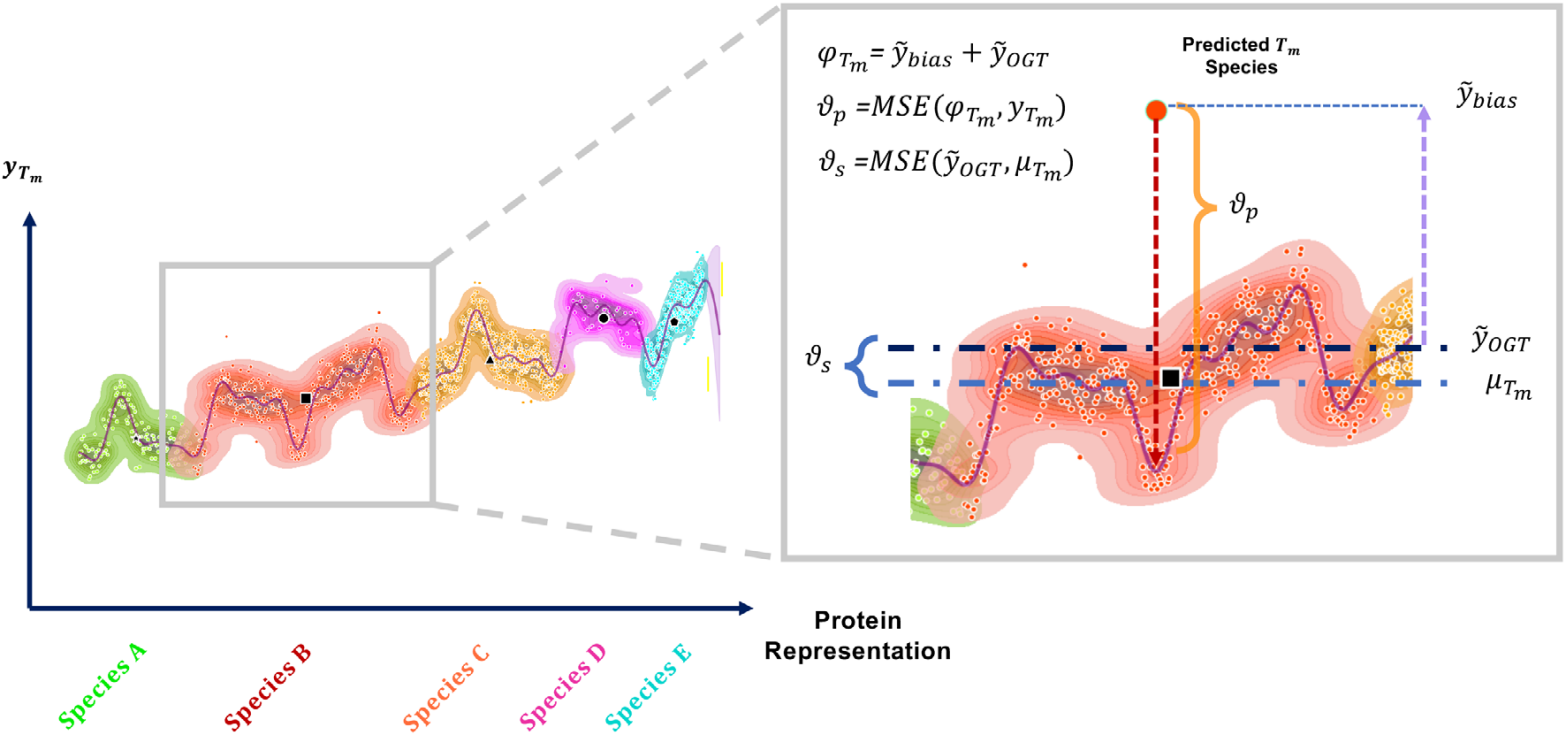
Schematic of the mechanism of action of the dual loss function, for simplicity depicting proteins along a 1‐dimensional axis. In the graph, the densities represent high probability regions for the fit to $T_{m}$ for each protein within the training set. The density is color‐coded to denote the species to which the points within that density belongs. The composition of the dual loss is depicted in the highlighted region. Here, $\varphi_{T_{m}}$ represents the predicted $T_{m}$, calculated as the sum of ${\tilde{y}}_{\mathrm{OGT}}$ (predicted OGT) and ${\tilde{y}}_{\text{bias}}$ (predicted bias), and $\mu_{T_{m}}$ denotes the species‐specific mean $T_{m}$. The term $\vartheta_{p}$ denotes the MSE between $\varphi_{T_{m}}$ and the ground truth $T_{m}$ ($y_{T_{m}}$), while $\vartheta_{s}$ represents the MSE between ${\tilde{y}}_{\mathrm{OGT}}$ and the reference OGT ($\mu_{T_{m}}$). The dual loss is defined as the sum of $\vartheta_{p}$ and $\vartheta_{s}$.

To give an impression of how good these predictions are in absolute terms, we also include an even simpler reference baseline for the species‐specific models, where predictions merely consist of the mean melting temperature observed for that species in the training set (Figure 4a–e, vertical bars). It is remarkable that for many of the datasets, the gains obtained with state‐of‐the‐art modeling procedures relative to this very simplistic baseline are minimal, and that the RMSEs that we obtain for these methods are almost as high as the standard deviations in the individual test sets (corresponding to the RMSE of the mean predictor).

## 3 Discussion

The results of this paper are somewhat sobering: the performance of melting temperature prediction is substantially lower than what the current literature suggests, and current approaches to task‐specific learning and fine‐tuning struggle to learn features that generalize across species. There are a few caveats to these conclusions. First, the high Spearman rhos reported in the literature do reflect an ability to predict the effect on melting temperature on the overall amino acid composition. While they are not sensitive to the effect of individual mutations, we might reasonably expect that there is sufficient signal for e.g., de novo design protocols to design toward specific melting temperature profiles. Secondly, while we in this paper focus our attention on *supervised* learning strategies, it would be worth investigating how unsupervised models fare on this problem, specifically in light of the high performances reported on absolute free energy prediction [9]. Finally, while we have tested a broad selection of methods, we have by no means exhaustively explored the space of modeling strategies. In particular, it is likely that better ways exist to incorporate 3D structure into the models. The log‐likelihoods of inverse‐folding models are known to correlate well with protein stability [5, 28], and fine‐tuning such models might therefore prove to generalize better across species.

The trade‐off between predicting global and local properties of proteins is presumably not unique to melting temperature prediction. In the literature on variant effects, a distinction is sometimes made between global and local epistasis, where the former describes the genetic background in which variants occur, influencing the effects of observed point mutants [29]. From this perspective, the cross‐species models in our melting temperature experiments display an inclination toward capturing the diffuse effects of the genetic background rather than the specific effects of individual mutations. We might expect to see similar effects when predicting other protein properties, in particular those tied to specific/niche habitat conditions such as temperature, pH, or salinity. When designing predictors for such tasks, it is therefore important that we design our metrics and train/validation/test setups to faithfully reflect the real‐world application of interest. A notable positive example is found in the prediction of clinical variant effects in human populations, where substantial work has been done to document biases and temper overly optimistic performance estimates [30].

## 4 Materials and Methods

### 4.1 Embedding Types

Our model employs two different embedding types: a sequence‐based embedding from the ESM2 (Evolutionary Scale Modeling) model [19] and an embedding obtained from the inverse folding model PiFold [20]. The former is an unsupervised language model based on the transformer architecture, trained on 250 million sequences from the UniRef dataset, which has been shown to capture and learn relevant information such as biochemical properties of amino acids, residue contact mapping, and homology detection, making it well‐suited for various downstream tasks [19, 26, 31]. As an inverse folding method, PiFold aims to predict protein sequences based on the atomic coordinates of protein backbone structures, and thus more directly captures the structural environment in its embeddings.

### 4.2 Model Design

The fundamental structure of the models is illustrated in Figure 1. The sequence and structural embeddings produce per‐residue vector representations of 1280 and 128 dimensions, respectively. These were either used independently or concatenated per residue and passed through a light attention block (see Figure 1). As the name suggests, light attention is a light‐weight mechanism to approximate a traditional attention mechanism and is commonly used as an alternative to simpler aggregation options such as calculating a mean over the sequence length [23]. This approach allows a unified and efficient representation of protein features, regardless of sequence length.

The light attention block was implemented using the default parameters as described in [23], although the dimensionality of the input embeddings was adjusted based on the type of embedding used. For prediction, a multilayer perceptron (MLP) was used, comprising three hidden layers with an architecture of (128, 64, n), where n represents the number of output components, which varies depending on the specific experiment. This configuration allows for model adaptability to different predictive tasks while maintaining a consistent base structure.

### 4.3 Rank‐N‐Contrast

The Rank‐N‐Contrast [21] procedure consists of 3 components:

- Data augmentation: A batch of input‐label pairs undergoes data augmentation in the original embedding space using a Gaussian perturbation. While the original description of the method proposed constructing a two‐view batch consisting of two types of augmentations, we deviate slightly from this by creating a two‐view batch which comprises the original input and a transformed version of the same input.
- Rank‐N‐Contrast Loss: A regression‐aware loss function is constructed to learn a representation of the input data such that relative distances reflect differences in their continuous target values (i.e., melting temperatures). Following the original publication, the R
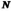
C loss per sample is defined as:

$$l_{\mathrm{RNC}}^{\left(i\right)}=\frac{1}{2N-1}\sum_{j=1,j\neq i}^{2N}-\log\mathbb{P}\left(v_{j}|v_{i},S_{i,j}\right)$$

where:

$$\mathbb{P}\left(v_{j}|v_{i},S_{i,j}\right)=\frac{\exp\left(\mathrm{sim}\left(v_{i},v_{j}\right)/\tau \right)}{\sum_{v_{k}\in S_{i,j}}\exp\left(\mathrm{sim}\left(v_{i},v_{k}\right)/\tau \right)}$$

Here, $\mathrm{sim}\left(\cdot ,\cdot \right)$ is a similarity in representation‐space between samples $v_{i}\text{and}v_{j}$, and τ is a temperature parameter. $\mathbb{P}\left(v_{j}|v_{i},S_{i,j}\right)$ represents the likelihood of a sample $v_{j}$ in the context of anchor $v_{i}$, and the set of samples $S_{i,j}$ that relative to $v_{i}$ have a worse rank than $v_{j}$ in terms of the *output* values.

$$S_{i,j}≔\left\{v_{k}|k\neq i,d\left({\tilde{y}}_{i},{\tilde{y}}_{k}\right)\geq d\left({\tilde{y}}_{i},{\tilde{y}}_{j}\right)\right\}$$

As an example, if $v_{j}$ is the sample that is closest to $v_{i}$ in terms of melting temperature, the likelihood would be optimized when $v_{j}$ had the highest similarity to $v_{i}$ in representation space. The full likelihood is simply an average over all samples in the batch.

$${ℒ}_{\mathrm{RNC}}=\frac{1}{2N}\sum_{i=1}^{2N}l_{\mathrm{RNC}}^{\left(i\right)}$$

- Representation Learning: The model learns an encoder by optimizing ${ℒ}_{\mathrm{RNC}}$. Once the representation is learned, the encoder is frozen, and a separate predictor is trained using a standard loss. In our case, the learned Rank‐N‐Contrast representations are used as direct input to the light attention block in combination with MLP block in Figure 1.

### 4.4 Data

All analyses were done on the Meltome partition of the FLIP dataset [12]. The dataset comprises 15 partitions designed to address various tasks in the field of protein design. For this study, we specifically focused on the partition related to thermostability, based on proteins with their corresponding melting temperatures ($T_{m}$) derived from the Meltome Atlas database. The “Mix” partition was selected, characterized by a high diversity of proteins originating from multiple species. Proteins less than 50 residues in length were discarded for this work. The number of proteins corresponding to each species in the splits of the FLIP dataset is presented in the Table 2.

**TABLE 2:** Sample distribution table by species.

Species	Sample size		
	Train	Val	Test
*Mus musculus* BMDC lysate	3108	369	227
HAOEC	2157	242	488
*Caenorhabditis elegans* lysate	1900	195	350
*Mus musculus* liver lysate	1308	139	71
*Escherichia coli* cells	1254	133	49
*Arabidopsis thaliana* seedling lysate	1252	139	203
*Saccharomyces cerevisiae* lysate	1245	123	226
Ecoli Lysate	1108	137	212
*Bacillus subtilis* 168 lysate R1	931	91	158
*Drosophila melanogaster* SII lysate	895	103	152
HEK293T	735	76	123
HepG2	647	71	85
HL60	628	79	82
*Thermus thermophilus* HB27 cells	573	59	58
*Oleispira antarctica* RB 8 lysate R1	555	77	125
*Geobacillus stearothermophilus* NCA26 lysate	541	68	36
*Picrophilus torridus* DSM9790 lysate	539	53	138
Jurkat	499	53	49
colon cancer spheroids	486	50	74
*Thermus thermophilus* HB27 lysate	426	47	105
U937	380	29	16
HaCaT	293	42	33
K562	237	28	2
pTcells	185	24	3
*Danio rerio* Zenodo lysate	119	11	11

*Note*: This table was constructed based on the distribution provided by the flip partition in relation to the Meltome Atlas, using the cross‐species partition or “mix”.

Protein sequences from the FLIP splits were used to compute embeddings using ESM2, which were then stored in files for efficient loading during model induction/inference. To map structural features, the UniProtIDs of each protein were used as a query in AlphaFoldDB [32] to obtain predicted structures. This approach was adopted to ensure the use of the closest predicted protein structure for each sequence, while avoiding the complexity associated with multi‐chain protein structures. Once the protein structures were obtained, PiFold was used to generate graph embeddings, which were then stored in files. Notably, only the encoder component of the algorithm was used.

### 4.5 Training

Model parameters were optimized using AdamW [33]. The learning rate (lr) was adjusted according to the type of experiment: for training on the full FLIP training partition in the global model setting (all species in the dataset), we used $\mathrm{lr}=1\times 10^{-4}$, while for the training of species‐specific models (individual models trained with proteins belonging to the same species), $\mathrm{lr}=1\times 10^{-3}$ was used.

Due to its particular architecture, the Rank N Contrast model followed a two‐stage procedure, first learning representations using a contrastive procedure and then learning a regressor from these representations. The two components were optimized with different learning rates, denoted as ${\mathrm{lr}}_{e}$ (encoder) and ${\mathrm{lr}}_{p}$ (decoder). For global model calibration, ${\mathrm{lr}}_{e}=1\times 10^{-5}$ and ${\mathrm{lr}}_{p}=1\times 10^{-4}$, while for species‐specific models, ${\mathrm{lr}}_{e}=1\times 10^{-5}$ and ${\mathrm{lr}}_{p}=1\times 10^{-3}$.

The fine‐tuning approach was run exactly as originally described [22], following the provided Jupyter notebooks.

## Author Contributions

**Sebastián García López:** conceptualization, investigation, methodology, software, writing – original draft, validation, writing – review and editing. **Jesper Salomon:** conceptualization, investigation, validation, supervision, writing – review and editing. **Wouter Boomsma:** conceptualization, methodology, investigation, validation, supervision, writing – original draft, writing – review and editing.

## Disclosure

The authors have nothing to report.

## Conflicts of Interest

The authors declare no conflicts of interest.

## Supporting information


**Data S1.** Supplementary Information.

## Data Availability

Data sharing is not applicable to this article as no new data were created or analyzed in this study.
