# Supplementary material for: Supervised Learning of Protein Melting Temperature: Cross‐Species vs. Species‐Specific Prediction
Source: Proteins. 2025 Jul 14;93(12):2158–66. doi: 10.1002/prot.70019 (PMC12594180; doi:10.1002/prot.70019)
Supplement: Supplementary file 1 — Data S1. Supplementary Information. [file PROT-93-2158-s001.pdf]

SUPERVISED LEARNING OF PROTEIN MELTING TEMPERATURE: CROSS-SPECIES VS SPECIES-SPECIFIC PREDICTION

SUPPLEMENTARY MATERIAL

Sebastián García López
Department of Computer Science - DIKU
University of Copenhagen
Copenhagen, Denmark

Jesper Salomon
Enzyme Research Division,
Novonesis A/S,
Kongens Lyngby, Denmark

Wouter Boomsma
Department of Computer Science - DIKU
University of Copenhagen
Copenhagen, Denmark
wb@di.ku.dk

Methods	balanceMSE				Dual loss				MSE			
	Spearman	MSE	RMSE	MAE	Spearman	MSE	RMSE	MAE	Spearman	MSE	RMSE	MAE
Arabidopsis_thaliana_seedling_lysate	-0.0988	56.3553	7.5070	5.9166	0.1737	40.3051	6.3486	4.6455	0.1713	41.6821	6.4562	4.8311
Bacillus_subtilis_168_lysate_R1	-0.1196	102.9299	10.1454	8.4139	0.0487	65.4492	8.0901	5.8427	0.0586	66.5909	8.1603	6.0094
Caenorhabditis_elegans_lysate	0.1683	61.1264	7.8183	6.6306	0.2485	28.5346	5.3418	4.4649	0.3400	31.8574	5.6442	4.7308
Danio_rerio_Zenodo_lysate	0.0818	84.3226	9.1827	8.0521	0.7636	48.2345	6.9451	5.6205	0.3636	49.3300	7.0235	5.6581
Drosophila_melanogaster_SII_lysate	0.0362	49.3716	7.0265	5.8863	0.3217	14.5884	3.8195	3.0671	0.2870	22.8456	4.7797	3.9267
Ecoli_Lysate	0.1727	51.7253	7.1920	5.8725	0.2293	53.0866	7.2861	5.7959	0.2485	43.9449	6.6291	5.3792
Escherichia_coli_cells	0.5557	51.5221	7.1779	6.3236	0.4178	104.6730	10.2310	8.8138	0.4553	71.6161	8.4626	6.9848
Geobacillus_stearothermophilus_NCA26_lysate	0.3519	186.9303	13.6722	12.2979	0.2649	167.7061	12.9501	9.4482	0.3040	185.6414	13.6250	11.2403
HAOEC	0.0601	49.3427	7.0244	5.1104	0.5448	34.5538	5.8782	4.3843	0.5905	28.7833	5.3650	3.9630
HEK293T	0.0989	21.5179	4.6387	3.5816	0.5893	14.4708	3.8040	2.9393	0.5569	12.9611	3.6001	2.8180
HL60	0.2614	15.4970	3.9366	3.0002	0.5686	16.5823	4.0721	2.7250	0.6305	13.4951	3.6736	2.5821
HaCaT	0.1179	39.1102	6.2538	4.7219	0.5411	28.7117	5.3583	4.0400	0.5127	27.3408	5.2288	4.1923
HepG2	-0.0364	42.8814	6.5484	5.2415	0.6338	25.9643	5.0955	3.9699	0.6174	21.9505	4.6851	3.8385
Jurkat	0.2568	27.7194	5.2649	4.3830	0.5620	19.2444	4.3868	3.6055	0.6110	17.6694	4.2035	3.4090
K562	1.0000	9.2415	3.0400	2.7739	-1.0000	21.4709	4.6337	3.8368	-1.0000	33.9431	5.8261	4.5849
Mus_musculus_BMDC_lysate	-0.0792	18.0147	4.2444	3.1469	0.2714	19.0719	4.3671	2.9600	0.2640	18.5152	4.3029	3.0061
Mus_musculus_liver_lysate	0.1486	44.4799	6.6693	5.3624	0.1226	41.9219	6.4747	5.2450	0.0522	38.7330	6.2236	5.0271
Oleispira_antarctica_RB-8_lysate_R1	0.1053	67.0532	8.1886	6.9769	0.2248	21.8788	4.6775	3.9502	0.2023	32.1654	5.6715	4.6370
Picrophilus_torridus_DSM9790_lysate	0.1438	208.7763	14.4491	13.3615	0.2341	81.2010	9.0112	5.7109	0.1184	89.9399	9.4837	6.5683
Saccharomyces_cerevisiae_lysate	0.1898	21.3975	4.6257	3.5893	0.3053	26.9074	5.1872	3.9288	0.3373	20.7970	4.5604	3.4606
Thermus_thermophilus_HB27_cells	0.1255	357.9547	18.9197	17.3433	0.2166	115.2598	10.7359	8.2072	0.2481	129.5257	11.3809	9.0225
Thermus_thermophilus_HB27_lysate	0.3065	214.3999	14.6424	13.2952	0.3608	41.2089	6.4194	5.1294	0.3968	50.3902	7.0986	5.8390
U937	-0.0889	32.4743	5.6986	4.4815	0.6765	12.6116	3.5513	2.6062	0.8059	9.5045	3.0829	2.3054
colon_cancer_spheroids	-0.0208	24.8119	4.9812	3.9806	0.0078	23.8056	4.8791	3.8172	-0.0378	24.8760	4.9876	3.8913
pTcells	-0.5000	29.8796	5.4662	4.8890	1.0000	17.0161	4.1251	4.0651	0.5000	4.3833	2.0936	1.8125
MEANS	0.1295	74.7534	7.7726	6.5853	0.3331	43.3783	6.1468	4.7528	0.3054	43.5393	6.0899	4.7887

Methods	Rank-N-contrast				Fine-tuning				DSP			
	Spearman	MSE	RMSE	MAE	Spearman	MSE	RMSE	MAE	Spearman	MSE	RMSE	MAE
Arabidopsis_thaliana_seedling_lysate	0.0795	42.2389	6.4991	4.9576	0.0966	45.2820	6.7292	5.0794	0.7123	21.3513	4.6207	2.7725
Bacillus_subtilis_168_lysate_R1	-0.0120	54.0174	7.3496	5.6810	0.0060	86.1529	9.2819	6.8091	0.7198	17.5704	4.1917	2.5414
Caenorhabditis_elegans_lysate	0.2828	43.6121	6.6039	5.7153	0.2705	34.5066	5.8742	4.7528	0.6889	14.4261	3.7982	2.6584
Danio_rerio_Zenodo_lysate	0.7182	41.5056	6.4425	4.9886	0.5182	40.3509	6.3522	4.6954	0.4818	32.1115	5.6667	3.7482
Drosophila_melanogaster_SII_lysate	0.2742	28.5485	5.3431	4.5660	0.3006	21.3529	4.6209	3.6642	0.6774	6.2719	2.5044	1.7874
Ecoli_Lysate	0.2318	45.1501	6.7194	5.3140	0.2454	49.3532	7.0252	5.6634	0.1519	126.2085	11.2343	8.8707
Escherichia_coli_cells	0.4180	115.1692	10.7317	9.4907	0.4022	74.4027	8.6257	7.1952	0.6269	38.0582	6.1691	4.1292
Geobacillus_stearothermophilus_NCA26_lysate	0.3045	390.0066	19.7486	18.7062	0.0445	177.5260	13.3239	10.9550	0.1424	662.8829	25.7465	24.9885
HAOEC	0.5018	33.2474	5.7661	4.2156	0.5229	29.7402	5.4535	4.1545	0.5042	35.7071	5.9755	4.4543
HEK293T	0.4592	14.6691	3.8300	3.0526	0.5214	15.5523	3.9436	2.9743	0.4125	21.4378	4.6301	3.3712
HL60	0.5910	11.7356	3.4257	2.5935	0.5032	14.9823	3.8707	2.9765	0.2335	19.9052	4.4615	3.5391
HaCaT	0.3780	31.8441	5.6431	4.5837	0.4557	30.1024	5.4866	4.2347	0.4056	32.7590	5.7235	4.5631
HepG2	0.5724	25.8588	5.0852	4.0280	0.6493	20.9923	4.5817	3.4581	0.3464	29.0320	5.3881	4.3085
Jurkat	0.5221	19.1855	4.3801	3.6597	0.4829	19.2886	4.3919	3.7606	0.2813	32.5298	5.7035	4.2631
K562	1.0000	15.6871	3.9607	3.8239	-1.0000	13.0006	3.6056	3.3152	1.0000	24.2264	4.9220	4.9157
Mus_musculus_BMDC_lysate	0.2366	13.5068	3.6752	2.6633	0.2629	18.9926	4.3581	3.3502	0.5882	9.2507	3.0415	2.3536
Mus_musculus_liver_lysate	-0.0144	39.0666	6.2503	5.1510	-0.0184	49.7340	7.0522	5.6574	0.1606	39.5936	6.2923	5.1442
Oleispira_antarctica_RB-8_lysate_R1	0.2087	37.8013	6.1483	5.2765	0.1830	48.2381	6.9454	5.2767	0.5651	11.2318	3.3514	2.2398
Picrophilus_torridus_DSM9790_lysate	0.0982	211.4720	14.5421	12.2113	0.1772	79.4651	8.9143	5.8800	0.7251	38.7400	6.2241	3.9950
Saccharomyces_cerevisiae_lysate	0.3222	20.4986	4.5275	3.4832	0.3043	28.1249	5.3033	3.8758	0.7484	9.7467	3.1220	2.1187
Thermus_thermophilus_HB27_cells	0.1148	323.8524	17.9959	16.0593	0.1978	99.7963	9.9898	6.9547	0.2558	117.8941	10.8579	8.5964
Thermus_thermophilus_HB27_lysate	0.3073	226.4277	15.0475	13.5913	0.3351	48.7605	6.9829	5.3385	0.5275	51.6840	7.1892	5.5169
U937	0.3971	17.5221	4.1859	3.3232	0.3471	20.0388	4.4765	3.4778	0.3353	22.1423	4.7056	3.7901
colon_cancer_spheroids	0.0026	20.7494	4.5552	3.6538	0.1072	26.6304	5.1605	4.0446	0.0993	22.7920	4.7741	3.8288
pTcells	0.5000	11.8894	3.4481	3.4415	0.5000	16.0521	4.0065	3.9391	-1.0000	24.3035	4.9299	4.5725
MEANS	0.3398	73.4105	7.2762	6.1692	0.2566	44.3367	6.2543	4.8593	0.4156	58.4742	6.2089	4.9227

Table S1: Cross-species performance using sequence-based embeddings (ESM2): Performance evaluation was performed using the full training and validation sets of FLIP partitions related to thermostability to induce the model. Testing was performed on individual species within the FLIP test set. The table shows the evaluation based on four primary metrics: Spearman correlation, MSE, RMSE and MAE, for the different methods analysed in this study: balanceMSE, Dual loss, MSE, Rank-N-contrast and fine-tuning. We also include the DSP baseline results.

Methods	balanceMSE				Dual loss			
	Spearman	MSE	RMSE	MAE	Spearman	MSE	RMSE	MAE
Arabidopsis_thaliana_seedling_lysate	0.1978	43.6081	6.6036	5.1771	0.0682	47.5744	6.8974	5.3945
Bacillus_subtilis_168_lysate_R1	0.1186	64.2171	8.0136	6.2432	0.1013	90.2633	9.5007	7.0436
Caenorhabditis_elegans_lysate	0.1135	77.9945	8.8314	7.4103	0.1488	67.6063	8.2223	6.8556
Danio_rerio_Zenodo_lysate	0.1545	67.2587	8.2011	5.6998	0.4818	85.5648	9.2501	7.8557
Drosophila_melanogaster_SII_lysate	0.1883	42.6002	6.5269	5.436	0.0446	51.1427	7.1514	5.9902
Ecoli_Lysate	0.0712	61.3955	7.8355	6.194	0.101	72.936	8.5403	6.6497
Escherichia_coli_cells	0.2221	135.125	11.6243	10.0279	0.0373	176.004	13.2666	11.8437
Geobacillus_stearothermophilus_NCA26_lysate	0.088	498.251	22.3215	21.2174	0.0049	478.348	21.8712	19.6788
HAOEC	0.3888	49.0695	7.005	5.4388	0.3905	41.4828	6.4407	4.8899
HEK293T	0.3737	24.4279	4.9425	3.768	0.3315	21.264	4.6113	3.5963
HL60	0.2711	23.8911	4.8879	3.6236	0.3725	17.2556	4.154	3.2397
HaCaT	0.2216	45.1755	6.7213	4.7603	0.242	37.8045	6.1485	4.8963
HepG2	0.3273	38.7255	6.223	4.6599	0.4269	34.835	5.9021	4.8028
Jurkat	0.1976	34.0146	5.8322	4.6808	0.4273	25.7759	5.077	4.1718
K562	-1	4.0299	2.0075	1.5151	-1	18.3807	4.2873	4.287
Mus_musculus_BMDC_lysate	0.1254	26.6945	5.1667	3.6526	0.1506	27.9881	5.2904	3.6731
Mus_musculus_liver_lysate	0.1326	46.4017	6.8119	5.5285	0.1947	46.4456	6.8151	5.6925
Oleispira_antarctica_RB-8_lysate_R1	0.1121	59.6047	7.7204	6.3074	0.0632	61.3767	7.8343	6.0074
Picrophilus_torridus_DSM9790_lysate	-0.041	410.771	20.2675	18.9768	0.126	305.723	17.4849	14.515
Saccharomyces_cerevisiae_lysate	0.053	32.4263	5.6944	4.5244	0.3205	29.4371	5.4256	4.2492
Thermus_thermophilus_HB27_cells	-0.1055	838.375	28.9547	26.1275	-0.0737	642.825	25.354	20.9251
Thermus_thermophilus_HB27_lysate	0.0468	582.313	24.1312	21.609	-0.0441	483.995	21.9999	17.1478
U937	0.3706	25.0477	5.0048	4.2869	0.1	30.4037	5.514	4.1933
colon_cancer_spheroids	0.0613	26.1402	5.1127	4.1275	0.2479	38.4994	6.2048	4.5201
pTcells	0.5	34.794	5.8986	5.8971	-0.5	50.3598	7.0965	6.875
MEANS	0.127576	131.694	9.29361	7.8756	0.110548	119.332	9.21362	7.55976

Methods	MSE				Rank-N-contrast			
	Spearman	MSE	RMSE	MAE	Spearman	MSE	RMSE	MAE
Arabidopsis_thaliana_seedling_lysate	0.0615	49.9119	7.0648	5.4569	0.04	47.601	6.8993	5.3863
Bacillus_subtilis_168_lysate_R1	0.0577	77.1768	8.785	6.8443	-0.0649	67.5899	8.2213	6.6734
Caenorhabditis_elegans_lysate	0.2483	88.5995	9.4127	8.0938	-0.0366	76.9103	8.7699	7.6532
Danio_rerio_Zenodo_lysate	-0.0182	63.064	7.9413	5.9462	0.6545	53.9727	7.3466	5.702
Drosophila_melanogaster_SII_lysate	0.2405	56.1757	7.495	6.493	0.3073	40.6112	6.3727	5.6224
Ecoli_Lysate	0.0873	66.5738	8.1593	6.4321	-0.0549	59.2102	7.6948	6.0148
Escherichia_coli_cells	0.0935	141.508	11.8957	10.15	0.0313	108.28	10.4057	9.1288
Geobacillus_stearothermophilus_NCA26_lysate	-0.0762	402.561	20.0639	18.2502	-0.2718	397.585	19.9395	18.0143
HAOEC	0.389	37.326	6.1095	4.579	0.3736	47.5098	6.8927	5.0855
HEK293T	0.3936	23.1623	4.8127	3.5236	0.3369	23.2582	4.8227	3.5999
HL60	0.3305	19.1996	4.3817	3.4348	0.354	19.2183	4.3839	3.1957
HaCaT	0.0785	40.7701	6.3851	5.359	0.3414	39.0351	6.2478	4.8033
HepG2	0.5175	25.7657	5.076	4.0447	0.4026	34.4372	5.8683	4.7732
Jurkat	0.5916	17.522	4.1859	3.4371	0.2134	31.7117	5.6313	4.5822
K562	1	5.6946	2.3863	2.1568	0	5.059	2.2492	1.8709
Mus_musculus_BMDC_lysate	0.21	20.926	4.5745	3.4033	0.1203	16.1929	4.024	2.9002
Mus_musculus_liver_lysate	-0.0108	46.809	6.8417	5.7137	-0.0597	48.1737	6.9407	5.7201
Oleispira_antarctica_RB-8_lysate_R1	0.0712	74.8146	8.6495	7.3418	0.1333	72.2167	8.498	7.3424
Picrophilus_torridus_DSM9790_lysate	0.1537	314.616	17.7374	15.7521	-0.0237	393.507	19.837	18.6257
Saccharomyces_cerevisiae_lysate	0.1838	21.5273	4.6398	3.6465	0.1058	21.5062	4.6375	3.6898
Thermus_thermophilus_HB27_cells	-0.1581	815.522	28.5573	25.5008	-0.013	860.323	29.3313	26.241
Thermus_thermophilus_HB27_lysate	0.0875	492.982	22.2032	19.2164	0.0335	609.46	24.6873	22.0347
U937	0.4765	22.0209	4.6926	3.7639	0.3316	28.9023	5.3761	4.2117
colon_cancer_spheroids	0.3813	23.1553	4.812	3.6682	0.27	18.2909	4.2768	3.5334
pTcells	0.5	46.0152	6.7835	6.4249	-0.5	36.0084	6.0007	5.9248
MEANS	0.235608	119.736	8.94586	7.54532	0.120996	126.263	9.0142	7.69319

Table S2: Cross-species performance using embeddings from protein structures via inverse folding (PiFold): Performance evaluation was performed using the full training and validation sets of FLIP partitions related to thermostability to induce the model. Testing was performed on individual species within the FLIP test set. The table shows the evaluation based on four primary metrics: Spearman correlation, MSE, RMSE and MAE, for the different methods analysed in this study: balanceMSE, Dual loss, MSE and Rank-N-contrast.

Methods	balanceMSE				Dual loss			
	Spearman	MSE	RMSE	MAE	Spearman	MSE	RMSE	MAE
Arabidopsis_thaliana_seedling_lysate	-0.0328	44.9936	6.7077	5.1725	0.1137	42.7874	6.5412	5.0204
Bacillus_subtilis_168_lysate_R1	0.0251	48.0577	6.9324	5.6365	-0.0123	75.4061	8.6837	6.5255
Caenorhabditis_elegans_lysate	0.1034	45.9781	6.7807	5.8325	0.3285	32.324	5.6854	4.6984
Danio_rerio_Zenodo_lysate	0.6455	61.3115	7.8302	6.5264	0.6455	46.4755	6.8173	5.611
Drosophila_melanogaster_SII_lysate	0.1124	25.6896	5.0685	4.1189	0.3215	20.6017	4.5389	3.6341
Ecoli_Lysate	0.0858	50.1222	7.0797	5.7697	0.2931	43.929	6.6279	5.4187
Escherichia_coli_cells	0.3961	124.554	11.1604	9.8918	0.3531	72.1184	8.4923	7.3403
Geobacillus_stearothermophilus_NCA26_lysate	0.2636	293.461	17.1307	15.6566	0.2785	131.69	11.4756	8.7325
HAOEC	-0.0117	52.6367	7.2551	5.5859	0.5974	28.9445	5.38	3.9445
HEK293T	-0.0166	30.0877	5.4852	4.3884	0.4751	16.0743	4.0093	3.0436
HL60	-0.026	27.6587	5.2592	4.0565	0.5124	13.6517	3.6948	2.713
HaCaT	-0.2289	55.3317	7.4385	5.8553	0.6654	24.5686	4.9567	4.0428
HepG2	-0.0209	47.0074	6.8562	5.5277	0.5767	23.547	4.8525	3.8697
Jurkat	-0.2288	45.9317	6.7773	5.9716	0.5776	16.0434	4.0054	3.3378
K562	1	2.7174	1.6485	1.5705	-1	55.7615	7.4674	5.3456
Mus_musculus_BMDC_lysate	-0.0049	23.717	4.87	3.8992	0.2541	18.3161	4.2797	2.9783
Mus_musculus_liver_lysate	0.1471	43.8143	6.6192	5.4036	0.1062	38.0584	6.1692	4.8434
Oleispira_antarctica_RB-8_lysate_R1	0.1304	32.0531	5.6616	4.8655	0.1592	32.9704	5.742	4.7691
Picrophilus_torridus_DSM9790_lysate	0.0946	168.576	12.9837	10.7069	-0.0145	80.3387	8.9632	6.2426
Saccharomyces_cerevisiae_lysate	0.0423	30.4126	5.5148	4.3921	0.3267	18.9499	4.3531	3.2769
Thermus_thermophilus_HB27_cells	0.2346	276.846	16.6387	14.5587	0.1349	102.891	10.1435	7.6395
Thermus_thermophilus_HB27_lysate	0.3538	143.249	11.9687	10.2945	0.2823	52.6987	7.2594	5.6441
U937	0.2529	33.3963	5.779	4.7876	0.4147	14.9665	3.8687	3.0603
colon_cancer_spheroids	-0.1639	31.1899	5.5848	4.6067	0.0389	24.9092	4.9909	3.8851
pTcells	0.5	21.1659	4.6006	4.2683	-1	19.0944	4.3697	3.8958
MEANS	0.146124	70.3984	7.58526	6.37376	0.217148	41.8846	6.13471	4.78052

Methods	MSE				Rank-N-contrast			
	Spearman	MSE	RMSE	MAE	Spearman	MSE	RMSE	MAE
Arabidopsis_thaliana_seedling_lysate	0.109	39.2262	6.2631	4.8214	0.1274	36.2154	6.0179	4.5516
Bacillus_subtilis_168_lysate_R1	0.0245	59.9784	7.7446	5.7714	0.0426	37.9457	6.16	4.8815
Caenorhabditis_elegans_lysate	0.3299	32.1148	5.667	4.8481	0.2677	33.5518	5.7924	4.8652
Danio_rerio_Zenodo_lysate	0.6636	47.1145	6.864	5.3625	0.6909	67.0075	8.1858	6.6925
Drosophila_melanogaster_SII_lysate	0.2991	22.454	4.7386	3.8411	0.381	17.4374	4.1758	3.4377
Ecoli_Lysate	0.2094	47.4049	6.8851	5.5404	0.2948	52.1178	7.2193	5.7509
Escherichia_coli_cells	0.3589	99.9341	9.9967	8.4899	0.2228	144.307	12.0128	10.624
Geobacillus_stearothermophilus_NCA26_lysate	0.2144	258.657	16.0828	13.9394	0.271	464.474	21.5517	20.7134
HAOEC	0.5121	32.7875	5.726	4.1267	0.4289	45.9515	6.7788	5.0711
HEK293T	0.4918	14.5158	3.81	2.9418	0.3636	18.5763	4.31	3.312
HL60	0.5576	25.8021	5.0796	3.0008	0.4153	15.7443	3.9679	2.8836
HaCaT	0.4221	29.4583	5.4275	4.4413	0.2096	35.3295	5.9439	4.6046
HepG2	0.5828	31.2366	5.589	4.0038	0.5321	31.8896	5.6471	4.3951
Jurkat	0.5608	34.4767	5.8717	4.0023	0.426	25.9512	5.0942	4.2632
K562	-1	714.098	26.7226	19.1323	-1	6.7103	2.5904	2.1838
Mus_musculus_BMDC_lysate	0.266	16.9931	4.1223	3.0564	0.2084	14.4295	3.7986	2.7389
Mus_musculus_liver_lysate	0.0062	40.8254	6.3895	5.0758	0.0941	48.9111	6.9936	5.7595
Oleispira_antarctica_RB-8_lysate_R1	0.1337	24.9606	4.9961	4.1284	0.1489	27.9463	5.2864	4.5097
Picrophilus_torridus_DSM9790_lysate	0.0185	119.918	10.9507	8.2336	0.0208	338.396	18.3955	16.5375
Saccharomyces_cerevisiae_lysate	0.3261	20.3761	4.514	3.4657	0.2844	27.5199	5.2459	4.2572
Thermus_thermophilus_HB27_cells	0.073	170.008	13.0387	10.0611	0.0676	431.125	20.7636	18.904
Thermus_thermophilus_HB27_lysate	0.2913	120.471	10.9759	7.906	0.2938	307.381	17.5323	16.1797
U937	0.3941	38.377	6.1949	3.9725	0.6	19.7639	4.4457	3.4367
colon_cancer_spheroids	0.0681	33.1918	5.7612	4.0455	-0.0761	23.4299	4.8404	3.8038
pTcells	-0.5	239.848	15.487	10.3164	0.5	27.3404	5.2288	5.0902
MEANS	0.21652	92.5691	8.19594	6.18098	0.232624	91.9781	7.91915	6.7779

Table S3: Cross-species performance using concatenation of embeddings from ESM2 and PiFold: Performance evaluation was performed using the full training and validation sets of FLIP partitions related to thermostability to induce the model. Testing was performed on individual species within the FLIP test set. The table shows the evaluation based on four primary metrics: Spearman correlation, MSE, RMSE and MAE, for the different methods analysed in this study: balanceMSE, Dual loss, MSE and Rank-N-contrast.

Methods	Dual loss				MSE			
	Spearman	MSE	RMSE	MAE	Spearman	MSE	RMSE	MAE
Arabidopsis_thaliana_seedling_lysate	0.3915	28.9075	5.3766	4.0307	0.2757	31.0004	5.5678	4.3043
Bacillus_subtilis_168_lysate_R1	0.3267	23.8897	4.8877	3.8329	0.2119	26.0025	5.0993	3.9440
Caenorhabditis_elegans_lysate	0.4500	21.8980	4.6795	3.5624	0.3638	23.3912	4.8364	3.7381
Danio_rerio_Zenodo_lysate	-0.1273	225.5203	15.0173	12.0062	0.1636	113.3124	10.6448	8.9790
Drosophila_melanogaster_SII_lysate	0.2276	12.6765	3.5604	2.8362	0.3183	11.3899	3.3749	2.6573
Ecoli_Lysate	0.4971	33.2729	5.7683	4.6260	0.4357	35.4790	5.9564	4.8229
Escherichia_coli_cells	0.6143	28.6546	5.3530	4.3872	0.5606	32.7802	5.7254	4.8546
Geobacillus_stearothermophilus_NCA26_lysate	0.0798	35.8998	5.9916	4.8417	0.2564	31.6178	5.6230	4.5296
HAOEC	0.6677	17.5784	4.1927	3.1416	0.6832	16.7097	4.0877	3.2149
HEK293T	0.6461	11.0681	3.3269	2.6517	0.5759	12.5330	3.5402	2.7081
HL60	0.6226	9.6282	3.1029	2.5825	0.6247	8.6127	2.9347	2.4174
HaCaT	0.2811	39.1072	6.2536	4.9183	0.6781	23.5524	4.8531	3.6007
HepG2	0.0500	39.9588	6.3213	5.1588	0.4166	27.5064	5.2447	4.1980
Jurkat	0.3752	24.4667	4.9464	4.2259	0.5497	20.3893	4.5155	3.6091
K562	-1.0000	8.9454	2.9909	2.6240	-1.0000	20.0878	4.4819	3.5169
Mus_musculus_BMDC_lysate	0.3697	10.7146	3.2733	2.3409	0.3265	10.8000	3.2863	2.3875
Mus_musculus_liver_lysate	0.0819	32.2965	5.6830	4.6590	0.0940	31.4523	5.6082	4.6244
Oleispira_antarctica_RB-8_lysate_R1	0.0335	18.6379	4.3172	3.2358	0.0911	18.3544	4.2842	3.2036
Picrophilus_torridus_DSM9790_lysate	0.2599	24.9528	4.9953	3.9412	0.2220	29.4879	5.4303	4.3721
Saccharomyces_cerevisiae_lysate	0.3283	17.5996	4.1952	3.1873	0.2468	18.8400	4.3405	3.2997
Thermus_thermophilus_HB27_cells	0.1867	57.0766	7.5549	6.2085	0.1717	57.7222	7.5975	6.3657
Thermus_thermophilus_HB27_lysate	0.2217	36.9322	6.0772	4.8796	0.0034	50.3409	7.0951	5.8207
U937	0.6588	11.8820	3.4470	2.6935	0.0412	51.1650	7.1530	4.7931
colon_cancer_spheroids	0.1363	17.5956	4.1947	3.4264	0.1359	19.8405	4.4543	3.6356
pTcells	-0.5000	8.1613	2.8568	2.5818	0.5000	3.8066	1.9511	1.9092
MEANS	0.2352	31.8928	5.1345	4.1032	0.2779	29.0470	5.1075	4.0603

Methods	Rank-N-contrast				Fine-tuning			
	Spearman	MSE	RMSE	MAE	Spearman	MSE	RMSE	MAE
Arabidopsis_thaliana_seedling_lysate	0.1801	33.4706	5.7854	4.4929	0.3288	31.3899	5.6027	4.2486
Bacillus_subtilis_168_lysate_R1	-0.0782	26.5269	5.1504	3.9686	0.3241	24.2249	4.9219	3.7920
Caenorhabditis_elegans_lysate	0.4126	23.4481	4.8423	3.6256	0.3798	23.6787	4.8661	3.7725
Danio_rerio_Zenodo_lysate	0.4818	369.5213	19.2229	18.4969	-0.1000	46.9320	6.8507	4.8788
Drosophila_melanogaster_SII_lysate	0.1157	11.7702	3.4308	2.7413	0.3258	11.2861	3.3595	2.7488
Ecoli_Lysate	0.4253	42.1144	6.4896	5.3407	0.4729	35.0761	5.9225	4.7830
Escherichia_coli_cells	0.3895	70.6396	8.4047	7.2346	0.5031	37.0275	6.0850	4.8651
Geobacillus_stearothermophilus_NCA26_lysate	0.1179	34.9162	5.9090	4.8523	0.1483	37.9108	6.1572	4.9882
HAOEC	0.6621	20.0385	4.4764	3.2012	0.6628	17.2208	4.1498	3.0952
HEK293T	0.6298	12.7439	3.5699	2.8206	0.5911	12.2922	3.5060	2.8222
HL60	0.6328	12.4881	3.5338	2.8032	0.6178	7.0974	2.6641	2.1099
HaCaT	0.2961	81.8340	9.0462	7.3093	0.5025	25.8462	5.0839	4.2798
HepG2	0.7306	18.2769	4.2751	3.3763	0.7558	13.5487	3.6809	2.8851
Jurkat	0.5600	26.8047	5.1773	4.1779	0.6656	13.4617	3.6690	2.8600
K562	-1.0000	33.2189	5.7636	5.5281	-1.0000	20.3373	4.5097	3.2317
Mus_musculus_BMDC_lysate	0.0678	12.1015	3.4787	2.5353	0.1992	13.5265	3.6778	2.6504
Mus_musculus_liver_lysate	0.0873	29.0420	5.3891	4.4974	0.2522	29.3449	5.4171	4.5132
Oleispira_antarctica_RB-8_lysate_R1	-0.0215	109.5010	10.4643	8.8855	0.1792	19.0637	4.3662	3.2617
Picrophilus_torridus_DSM9790_lysate	0.0355	234.6153	15.3172	13.4480	0.2999	25.7026	5.0698	3.9694
Saccharomyces_cerevisiae_lysate	0.1777	17.2934	4.1585	3.3133	0.3546	15.7263	3.9656	2.8929
Thermus_thermophilus_HB27_cells	0.1418	50.6287	7.1154	5.9142	0.2408	55.2620	7.4338	6.2439
Thermus_thermophilus_HB27_lysate	0.1577	38.7479	6.2248	5.1955	0.2376	37.6393	6.1351	5.0247
U937	0.5824	23.3845	4.8357	3.8638	0.7235	9.8428	3.1373	2.6623
colon_cancer_spheroids	-0.0200	22.0879	4.6998	3.6528	-0.1307	28.6225	5.3500	4.2177
pTcells	1.0000	193.4740	13.9095	13.5656	1.0000	4.6753	2.1623	1.8325
MEANS	0.2706	61.9475	6.8268	5.7936	0.3414	23.8694	4.7198	3.7052

Table S4: Species-specific performance of methods employing sequence-based embeddings from ESM2: The models were trained independently on each species and subsequently tested on the corresponding FLIP test partition. Spearman correlation, mean square error (MSE), root mean square error (RMSE) and mean absolute error (MAE) are reported for the different methods analysed in this study: Dual loss, MSE, Rank-N-contrast, and Fine-tuning. For certain species, it was necessary to adjust the learning rate during training to ensure stability in the optimization process. Specifically, for models corresponding to Caenorhabditis elegans lysate (MSE method) and Thermus thermophilus HB27 cells (biasg method), a learning rate of $\text{lr} = 1 \times 10^{-4}$ was implemented, contrasting with the standard value ($\text{lr} = 1 \times 10^{-3}$) established for species-specific individual model training.

Methods	Dual loss			
	Spearman	MSE	RMSE	MAE
Arabidopsis_thaliana_seedling_lysate	0.2502	31.8047	5.6396	4.36
Bacillus_subtilis_168_lysate_R1	0.2005	26.9053	5.187	4.0578
Caenorhabditis_elegans_lysate	0.2717	24.0761	4.9067	3.7928
Danio_rerio_Zenodo_lysate	-0.1909	85.0409	9.2218	6.6883
Drosophila_melanogaster_SII_lysate	0.2971	11.8738	3.4458	2.6509
Ecoli_Lysate	0.3893	38.8821	6.2355	5.0328
Escherichia_coli_cells	0.1184	53.963	7.346	6.2021
Geobacillus_stearothermophilus_NCA26_lysate	0.0842	41.817	6.4666	5.1216
HAOEC	0.5702	21.5063	4.6375	3.5582
HEK293T	0.3553	16.8959	4.1105	3.3724
HL60	0.5094	12.7012	3.5639	2.8331
HaCaT	0.0528	57.0223	7.5513	5.5844
HepG2	0.5148	25.9791	5.097	4.1233
Jurkat	0.0998	29.5164	5.4329	4.4984
K562	-1	14.7407	3.8394	3.6112
Mus_musculus_BMDC_lysate	0.2007	12.7979	3.5774	2.5749
Mus_musculus_liver_lysate	0.1861	139.129	11.7953	5.8553
Oleispira_antarctica_RB-8_lysate_R1	0.0777	17.7828	4.217	3.183
Picrophilus_torridus_DSM9790_lysate	0.113	28.2721	5.3171	4.3029
Saccharomyces_cerevisiae_lysate	0.1198	18.515	4.3029	3.395
Thermus_thermophilus_HB27_cells	-0.0566	63.1341	7.9457	6.5063
Thermus_thermophilus_HB27_lysate	0.1028	41.072	6.4087	5.3268
U937	0.1588	17.6512	4.2013	3.4349
colon_cancer_spheroids	0.0388	19.2596	4.3886	3.5495
pTcells	0.5	8.3234	2.885	2.6848
MEANS	0.158556	34.3465	5.50882	4.25203

Methods	MSE				Rank-N-contrast			
	Spearman	MSE	RMSE	MAE	Spearman	MSE	RMSE	MAE
Arabidopsis_thaliana_seedling_lysate	0.1513	33.8696	5.8198	4.4579	0.1197	32.8702	5.7332	4.5013
Bacillus_subtilis_168_lysate_R1	0.1527	28.1217	5.303	4.1673	0.147	26.1166	5.1104	4.0038
Caenorhabditis_elegans_lysate	0.3198	24.1727	4.9166	3.7621	0.1201	25.0226	5.0023	3.9697
Danio_rerio_Zenodo_lysate	0.6	33.9685	5.8282	4.6552	0.1545	39.9804	6.323	4.7511
Drosophila_melanogaster_SII_lysate	0.3204	10.97	3.3121	2.5842	0.3396	12.9318	3.5961	2.8986
Ecoli_Lysate	0.2583	42.3656	6.5089	5.2799	0.4019	48.3124	6.9507	5.6156
Escherichia_coli_cells	0.367	58.9055	7.675	6.5599	-0.0351	61.2983	7.8293	6.7248
Geobacillus_stearothermophilus_NCA26_lysate	-0.106	39.5683	6.2903	4.9254	0.1681	33.9255	5.8246	4.5943
HAOEC	0.6131	19.766	4.4459	3.5534	0.5151	24.2065	4.92	3.6582
HEK293T	0.5067	14.7227	3.837	3.0679	0.4558	14.4314	3.7989	2.998
HL60	0.5877	9.8337	3.1359	2.5131	0.1545	16.2364	4.0294	3.0754
HaCaT	0.4174	34.1148	5.8408	4.4338	0.3616	36.3923	6.0326	4.6482
HepG2	0.6413	19.7246	4.4412	3.4868	0.5916	23.1588	4.8124	3.8102
Jurkat	0.3163	26.6553	5.1629	4.2983	0.271	24.4258	4.9422	4.2085
K562	1	27.3812	5.2327	5.1922	-1	5.3537	2.3138	1.9069
Mus_musculus_BMDC_lysate	0.2036	12.4449	3.5277	2.4947	0.1087	11.8251	3.4388	2.535
Mus_musculus_liver_lysate	0.2019	26.4774	5.1456	4.1962	-0.0673	30.653	5.5365	4.6092
Oleispira_antarctica_RB-8_lysate_R1	0.0616	18.4959	4.3007	3.3799	0.1153	16.5094	4.0632	3.0985
Picrophilus_torridus_DSM9790_lysate	0.0001	31.7089	5.6311	4.547	0.2134	31.4108	5.6045	4.391
Saccharomyces_cerevisiae_lysate	0.2475	17.3746	4.1683	3.1798	0.2441	23.5091	4.8486	3.9765
Thermus_thermophilus_HB27_cells	-0.015	58.7667	7.6659	6.0328	-0.057	48.9728	6.9981	5.7465
Thermus_thermophilus_HB27_lysate	0.0471	49.1931	7.0138	5.7324	0.1022	37.4915	6.123	5.083
U937	-0.1235	24.0018	4.8992	3.9386	-0.1324	18.1745	4.2632	3.5649
colon_cancer_spheroids	0.0761	18.1357	4.2586	3.4174	0.0622	17.8032	4.2194	3.4283
pTcells	0.5	26.305	5.1288	4.9994	-0.5	18.0692	4.2508	3.7284
MEANS	0.293816	28.2818	5.1796	4.19422	0.114184	27.1633	5.0626	4.06104

Table S5: Species-specific performance of methods employing embeddings from protein structures using Inverse Folding algorithms (PiFold): The models were trained independently on each species and subsequently tested on the corresponding FLIP test partition. Spearman correlation, mean square error (MSE), root mean square error (RMSE) and mean absolute error (MAE) are reported for the different methods analysed in this study: Dual loss, MSE, and Rank-N-contrast.

Methods	Dual loss			
	Spearman	MSE	RMSE	MAE
Arabidopsis_thaliana_seedling_lysate	0.3321	30.1566	5.4915	4.1206
Bacillus_subtilis_168_lysate_R1	0.3015	26.9116	5.1876	4.2273
Caenorhabditis_elegans_lysate	0.4724	20.5988	4.5386	3.5272
Danio_rerio_Zenodo_lysate	0.3364	48.9019	6.993	5.3776
Drosophila_melanogaster_SII_lysate	0.4173	10.1633	3.188	2.5342
Ecoli_Lysate	0.5134	31.3316	5.5975	4.5145
Escherichia_coli_cells	0.5683	34.6805	5.889	4.8422
Geobacillus_stearothermophilus_NCA26_lysate	0.2924	31.5223	5.6145	4.5322
HAOEC	0.6785	16.2852	4.0355	3.166
HEK293T	0.5679	12.6298	3.5538	2.8093
HL60	0.663	9.4511	3.0743	2.5043
HaCaT	-0.3245	48.5773	6.9697	5.5694
HepG2	0.7914	14.4484	3.8011	3.0977
Jurkat	0.6486	16.1099	4.0137	3.2487
K562	-1	3.0531	1.7473	1.3394
Mus_musculus_BMDC_lysate	0.286	11.6081	3.4071	2.3945
Mus_musculus_liver_lysate	-0.0108	31.4768	5.6104	4.6453
Oleispira_antarctica_RB-8_lysate_R1	0.1266	16.7649	4.0945	3.0388
Picrophilus_torridus_DSM9790_lysate	0.0523	27.8212	5.2746	4.1684
Saccharomyces_cerevisiae_lysate	0.2702	16.1805	4.0225	3.0964
Thermus_thermophilus_HB27_cells	0.0171	55.0932	7.4225	5.989
Thermus_thermophilus_HB27_lysate	0.3609	38.8648	6.2342	5.0251
U937	0.6765	9.8029	3.131	2.6008
colon_cancer_spheroids	-0.1437	18.6022	4.313	3.4689
pTcells	-0.5	14.422	3.7976	3.5517
MEANS	0.255752	23.8183	4.6801	3.73558

Methods	MSE				Rank-N-contrast			
	Spearman	MSE	RMSE	MAE	Spearman	MSE	RMSE	MAE
Arabidopsis_thaliana_seedling_lysate	0.3461	29.1147	5.3958	4.1742	0.1444	33.0563	5.7495	4.4886
Bacillus_subtilis_168_lysate_R1	0.1653	25.9705	5.0961	4.0067	0.1319	25.9661	5.0957	3.9435
Caenorhabditis_elegans_lysate	0.499	20.6519	4.5444	3.4613	0.4784	21.4948	4.6362	3.4993
Danio_rerio_Zenodo_lysate	0.0455	109.125	10.4463	8.6692	0.0364	193.913	13.9253	12.4161
Drosophila_melanogaster_SII_lysate	0.3202	12.096	3.4779	2.7011	0.3287	10.5318	3.2453	2.5795
Ecoli_Lysate	0.4779	31.9304	5.6507	4.5741	0.5269	33.6137	5.7977	4.6787
Escherichia_coli_cells	0.5883	31.4554	5.6085	4.7336	0.5132	65.2721	8.0791	6.593
Geobacillus_stearothermophilus_NCA26_lysate	0.1593	37.1683	6.0966	5.0318	0.0914	33.9548	5.8271	4.8257
HAOEC	0.6695	16.8059	4.0995	3.148	0.6921	18.7967	4.3355	3.1634
HEK293T	0.4082	15.5122	3.9386	3.1006	0.5787	12.4972	3.5351	2.6919
HL60	0.6704	7.8528	2.8023	2.3577	0.6633	12.8484	3.5845	2.8722
HaCaT	0.4248	26.0739	5.1063	4.0582	-0.1043	150.318	12.2604	9.0783
HepG2	0.6422	20.104	4.4837	3.5313	0.7702	18.014	4.2443	3.3321
Jurkat	0.558	18.3289	4.2812	3.419	0.6849	23.2359	4.8204	3.8469
K562	1	42.885	6.5487	6.3414	-1	25.5311	5.0528	4.0778
Mus_musculus_BMDC_lysate	0.3303	11.1626	3.3411	2.47	0.1396	12.6878	3.562	2.5788
Mus_musculus_liver_lysate	0.1039	28.9951	5.3847	4.4282	0.2819	30.818	5.5514	4.3435
Oleispira_antarctica_RB-8_lysate_R1	0.0069	19.2697	4.3897	3.3043	0.2918	15.99	3.9988	2.9648
Picrophilus_torridus_DSM9790_lysate	0.2891	26.4809	5.146	4.1468	0.1515	137.6	11.7303	10.1876
Saccharomyces_cerevisiae_lysate	0.3907	15.1299	3.8897	2.9698	0.2702	16.3817	4.0474	3.1665
Thermus_thermophilus_HB27_cells	0.1968	58.7222	7.663	6.4591	0.1177	47.7578	6.9107	5.6658
Thermus_thermophilus_HB27_lysate	-0.1086	45.6133	6.7538	5.5957	0.0697	35.6751	5.9729	4.9608
U937	0.5059	12.1784	3.4898	3.0624	0.1618	29.4041	5.4226	4.2814
colon_cancer_spheroids	0.0397	18.7022	4.3246	3.4427	0.189	16.633	4.0784	3.201
pTcells	-0.5	9.1506	3.025	2.8475	0.5	198.42	14.0862	13.8163
MEANS	0.329176	27.6192	4.99936	4.08139	0.268376	48.8165	6.22198	5.09014

Table S6: Species-specific performance of methods employing the combination of ESM2 and PiFold embeddings: The models were trained independently on each species and subsequently tested on the corresponding FLIP test partition. Spearman correlation, mean square error (MSE), root mean square error (RMSE) and mean absolute error (MAE) are reported for the different methods analysed in this study: Dual loss, MSE, and Rank-N-contrast.

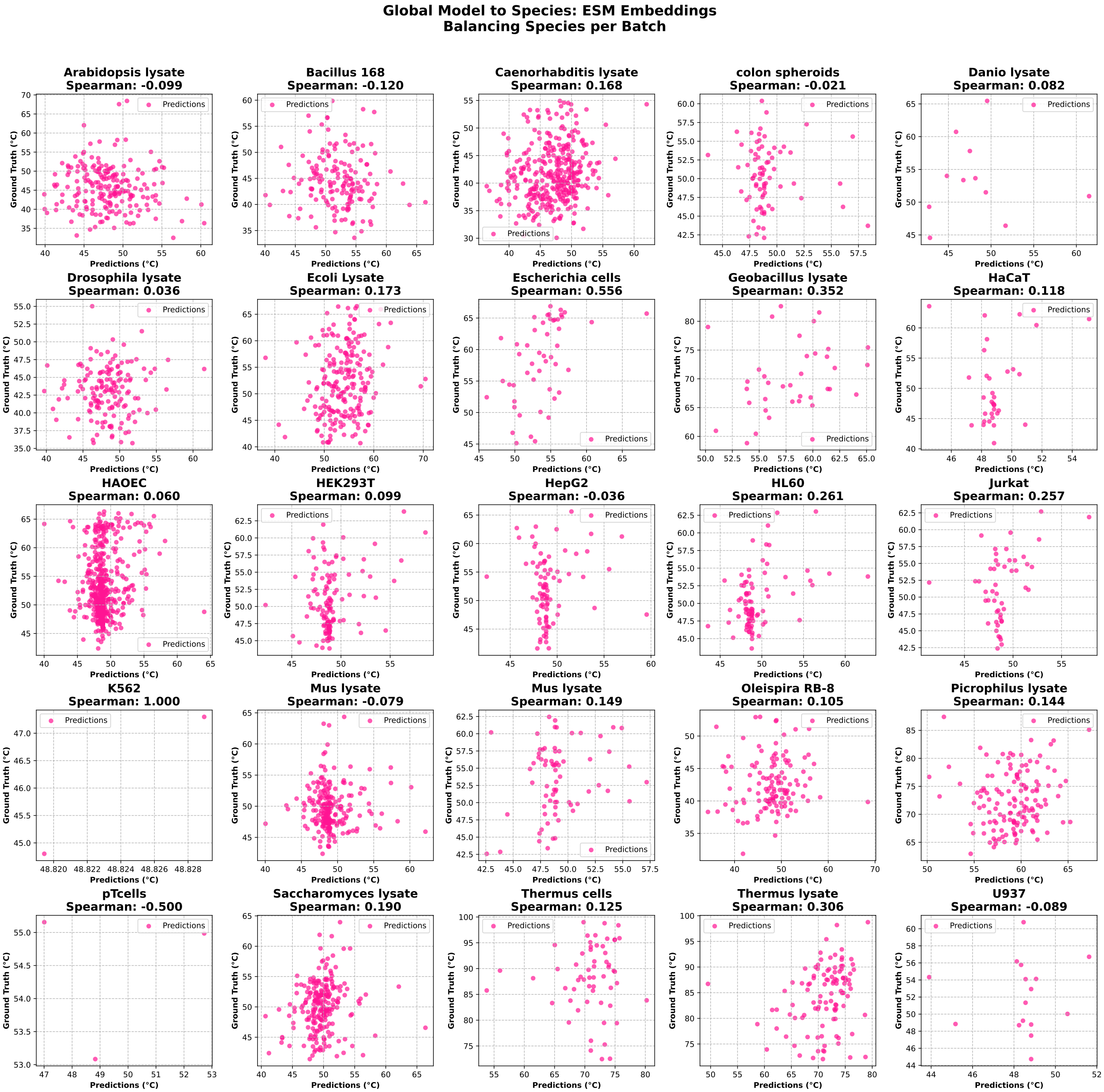

Figure S1: Scatterplots of the cross-species, balanceMSE setting: using a balancing strategy per batch and ESM embeddings.

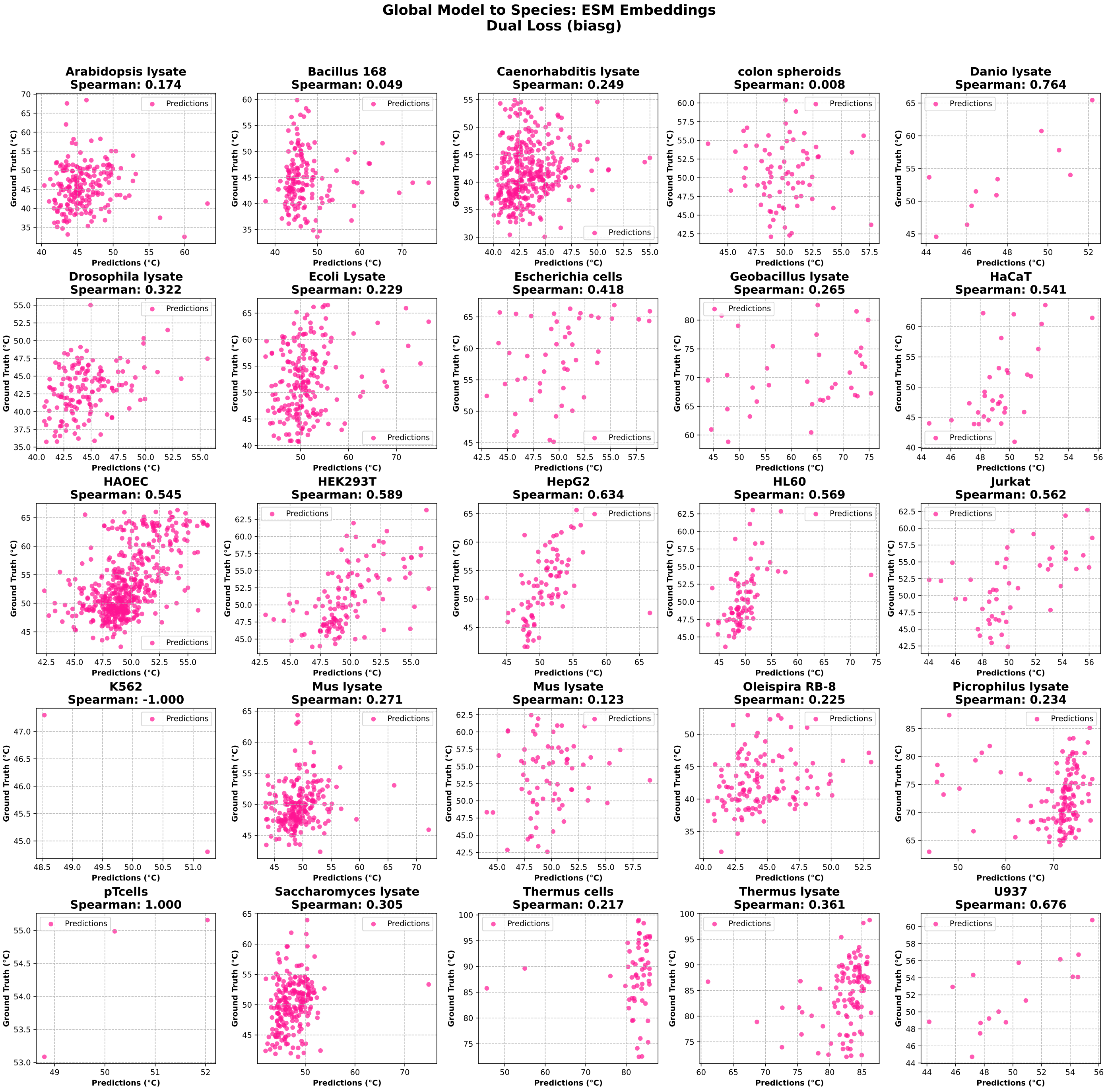

Figure S2: Scatterplots of the cross-species, dual-loss setting: using the dual loss function and ESM embeddings

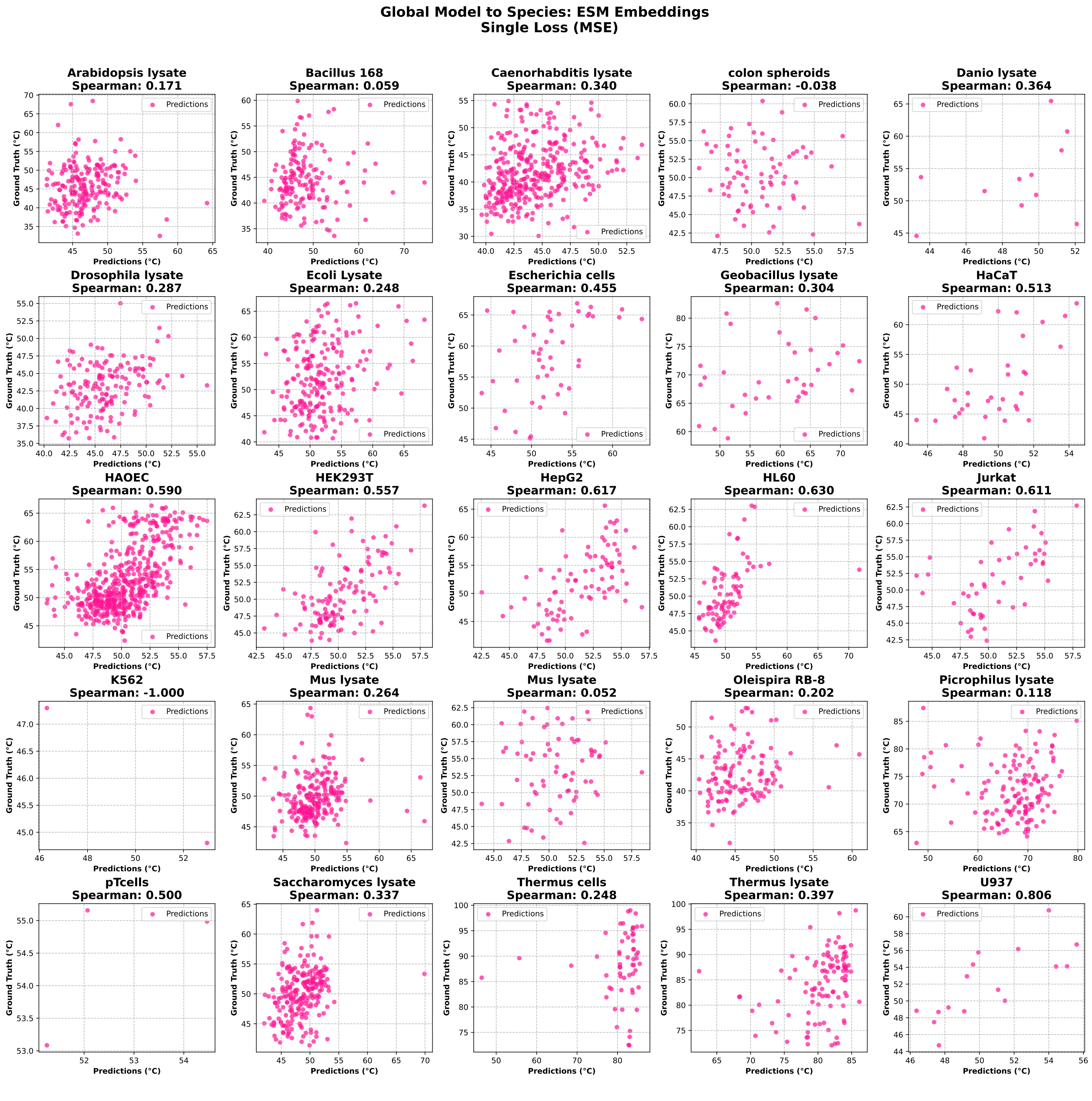

Figure S3: Scatterplots of the cross-species, MSE setting: a standard mean-squared error loss and ESM embeddings

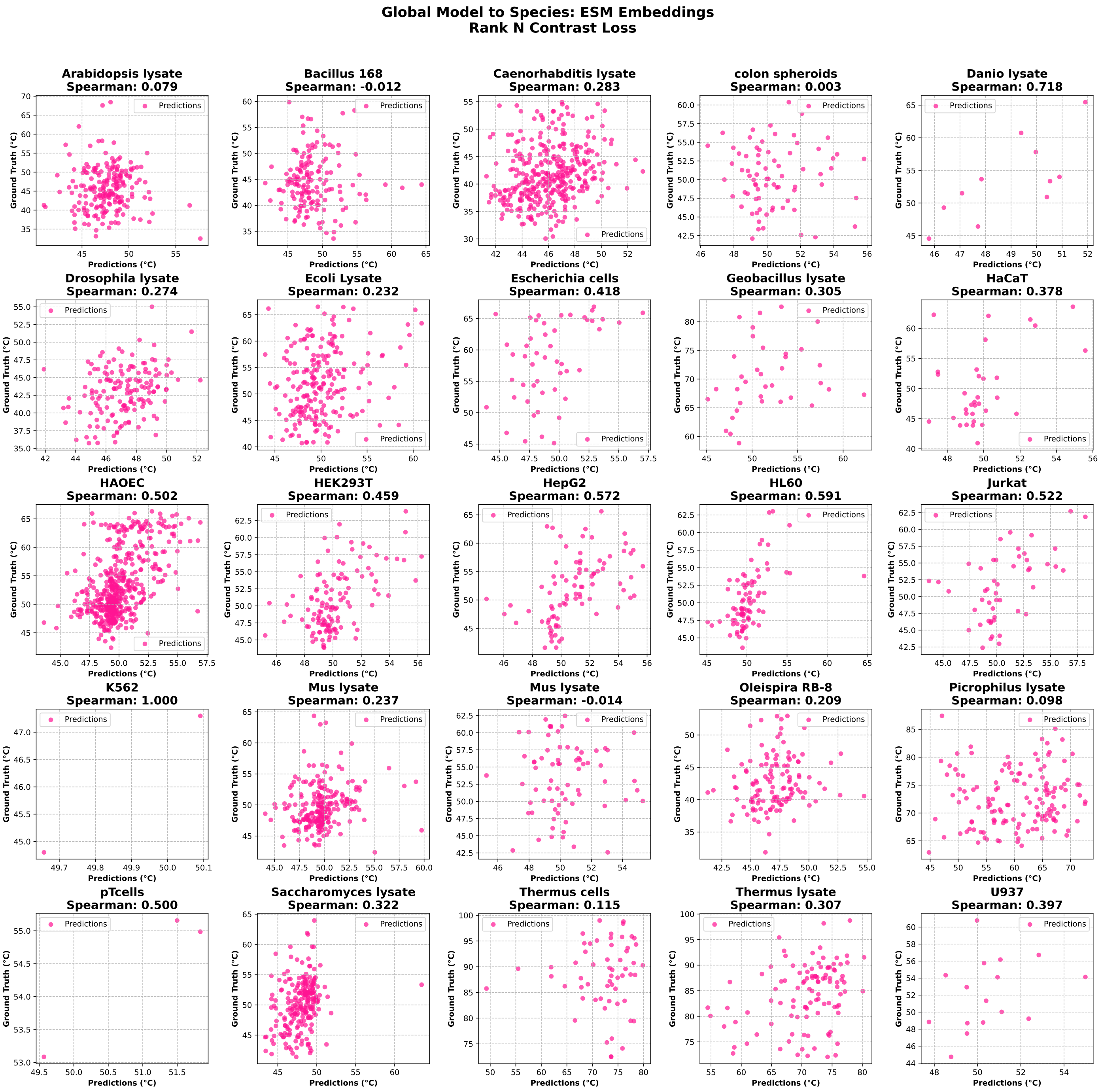

Figure S4: Scatterplots of the cross-species, rank-N-contrast setting: a contrastive representation learning approach and ESM embeddings

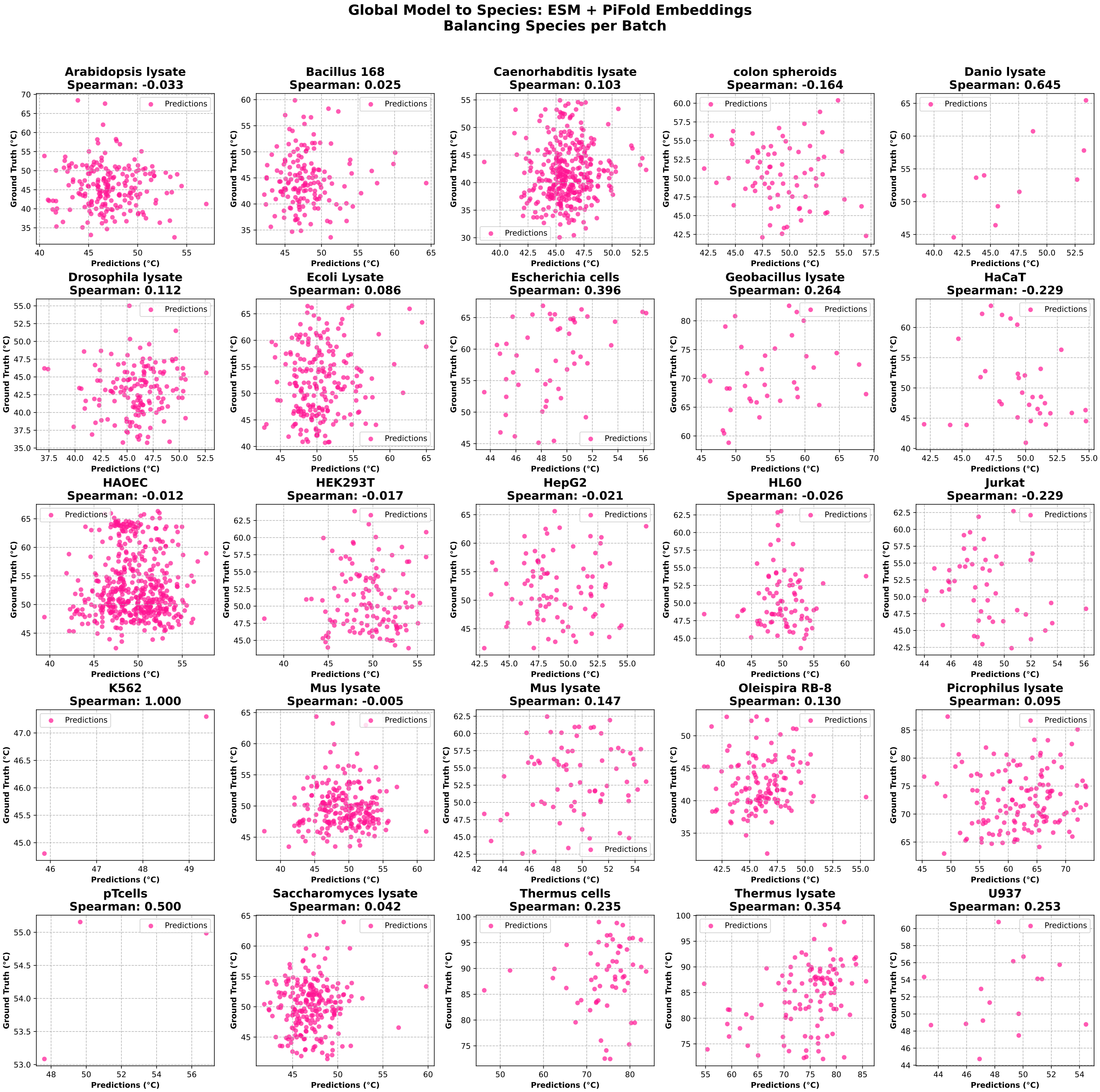

Figure S5: Scatterplots of the cross-species, balanceMSE setting: using a balancing strategy per batch in combination with a concatenation of ESM and PiFold embeddings

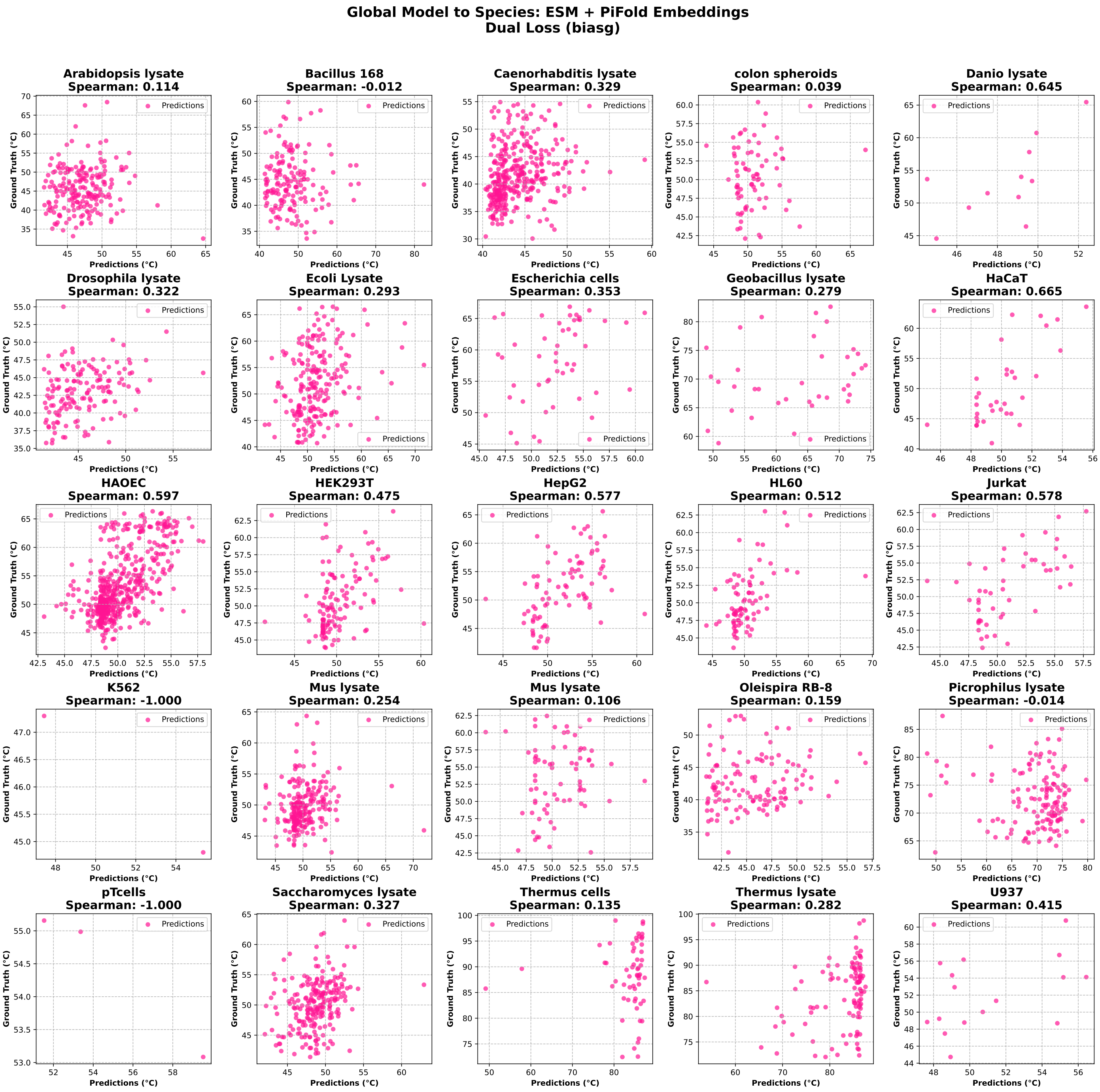

Figure S6: Scatterplots of the cross-species, dual-loss setting: using the dual loss function in combination with a concatenation of ESM and PiFold embeddings

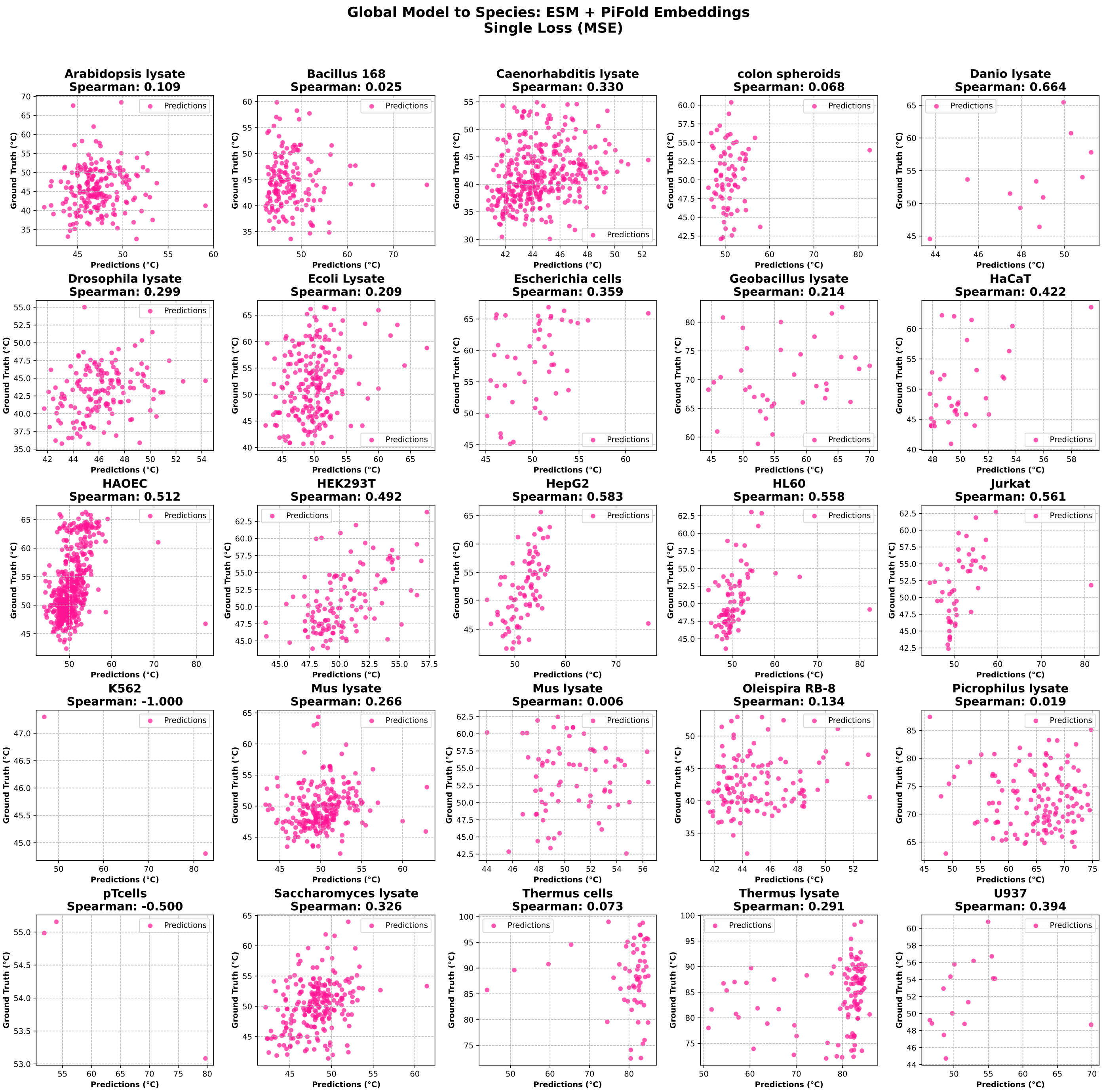

Figure S7: Scatterplots of the cross-species, MSE setting: a standard mean-squared error loss in combination with a concatenation of ESM and PiFold embeddings

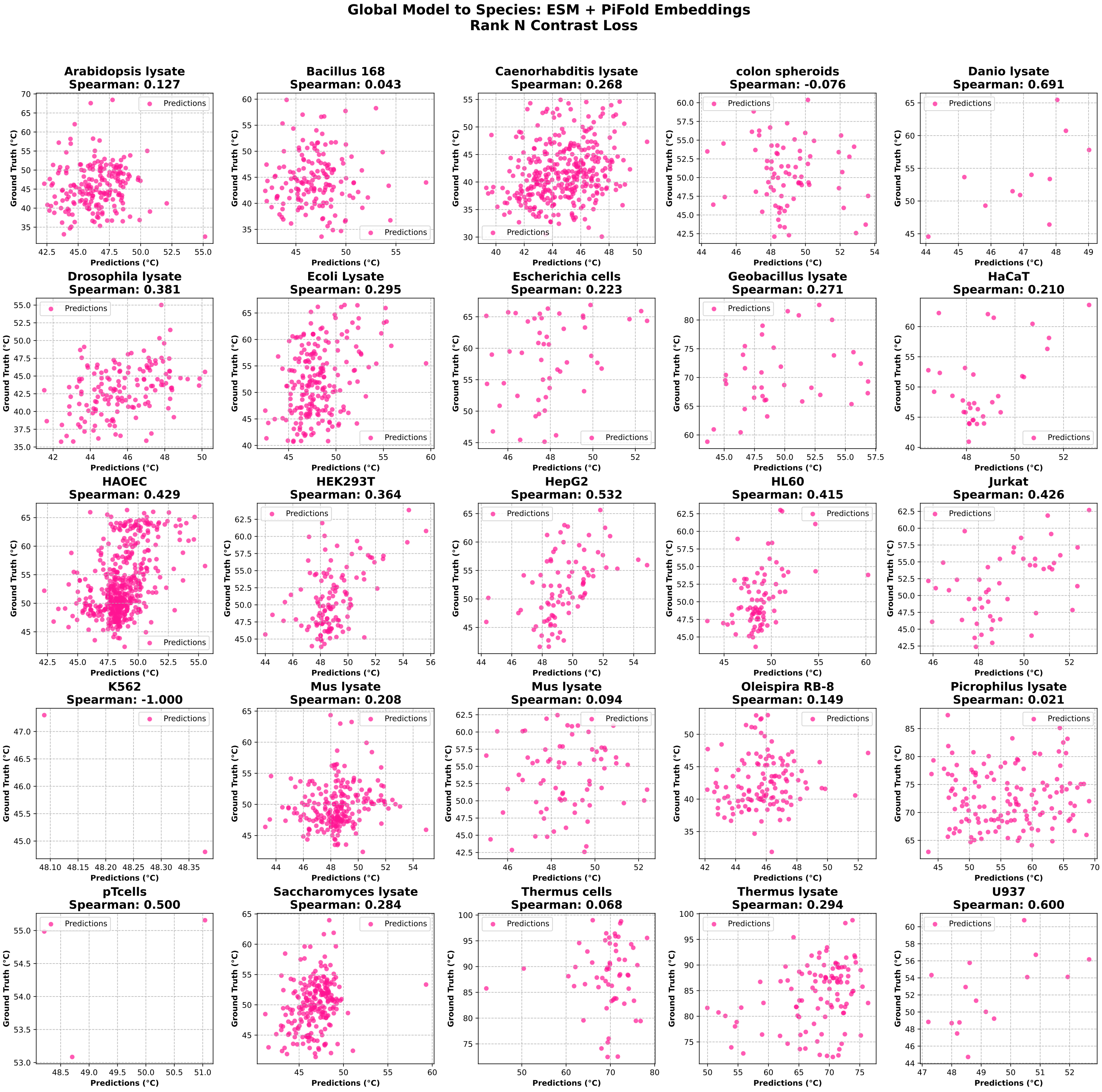

Figure S8: Scatterplots of the cross-species, rank-N-contrast setting: a contrastive representation learning approach in combination with a concatenation of ESM and PiFold embeddings

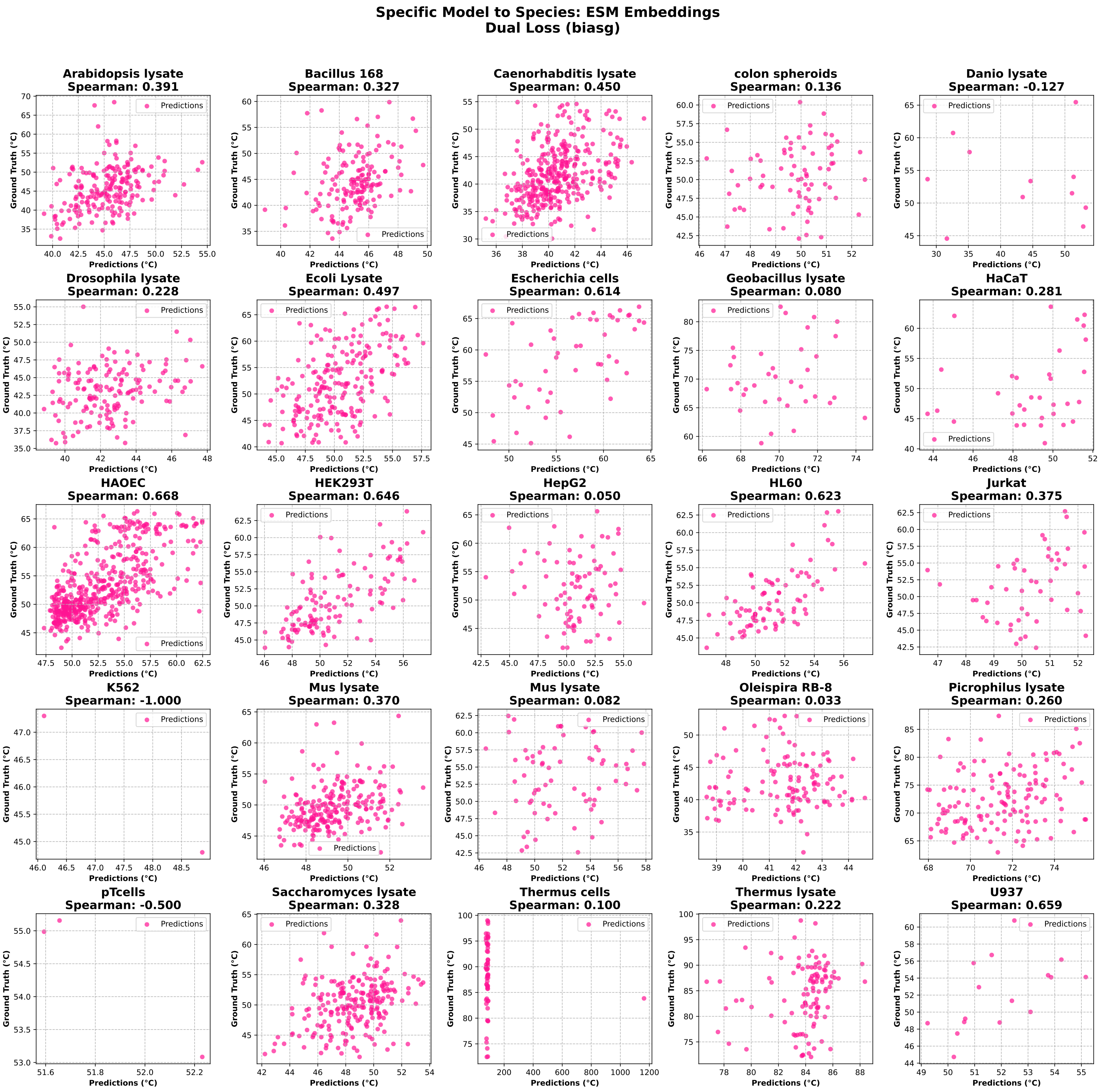

Figure S9: Scatterplots of the species-specific, dual-loss setting: using the dual loss function combined with ESM embeddings

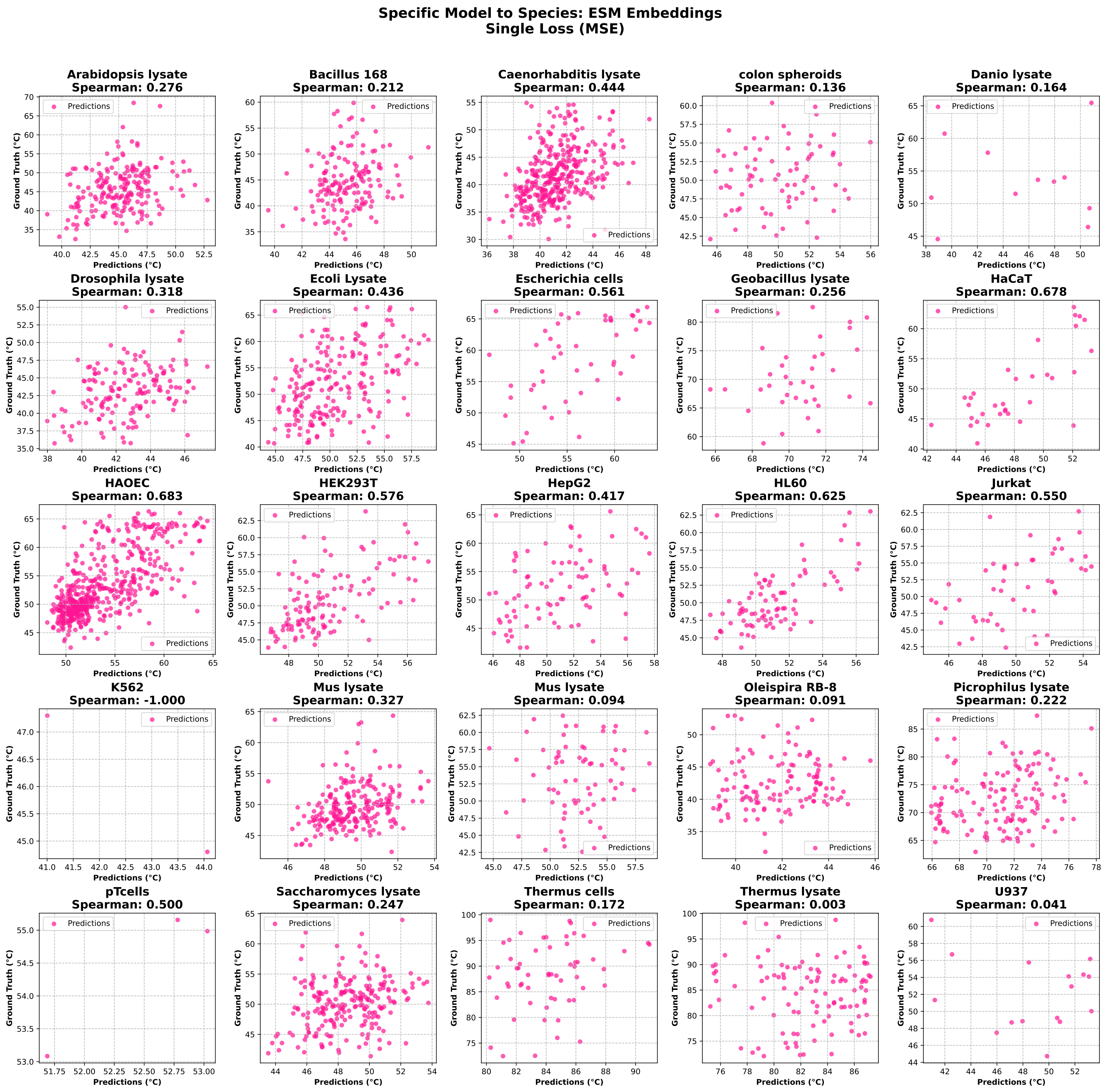

Figure S10: Scatterplot of the species-specific, MSE setting: a standard mean-squared error loss combined with ESM embeddings

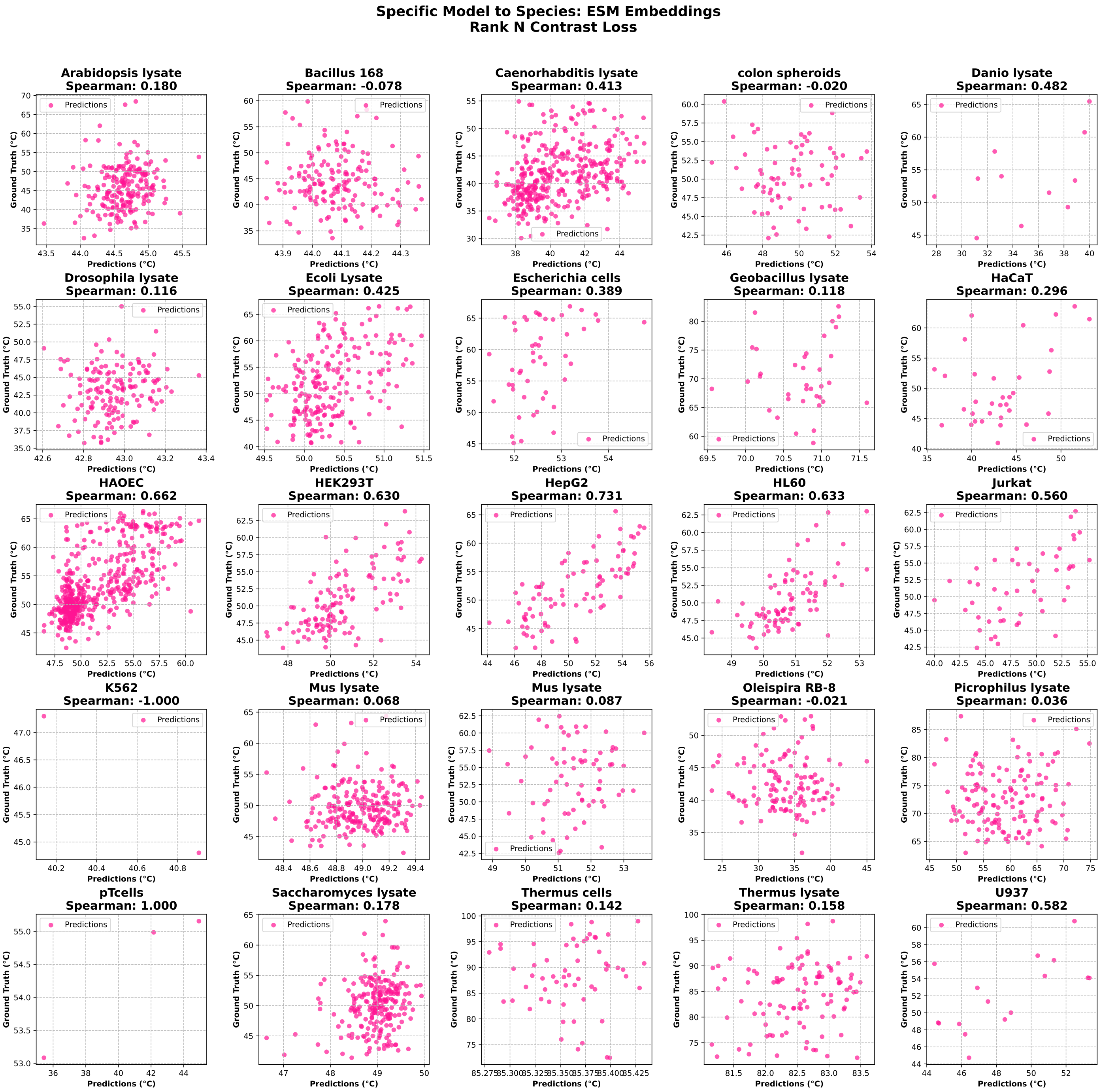

Figure S11: Scatterplot of the species-specific, rank-N-contrast setting: a contrastive representation learning approach combined with ESM embeddings

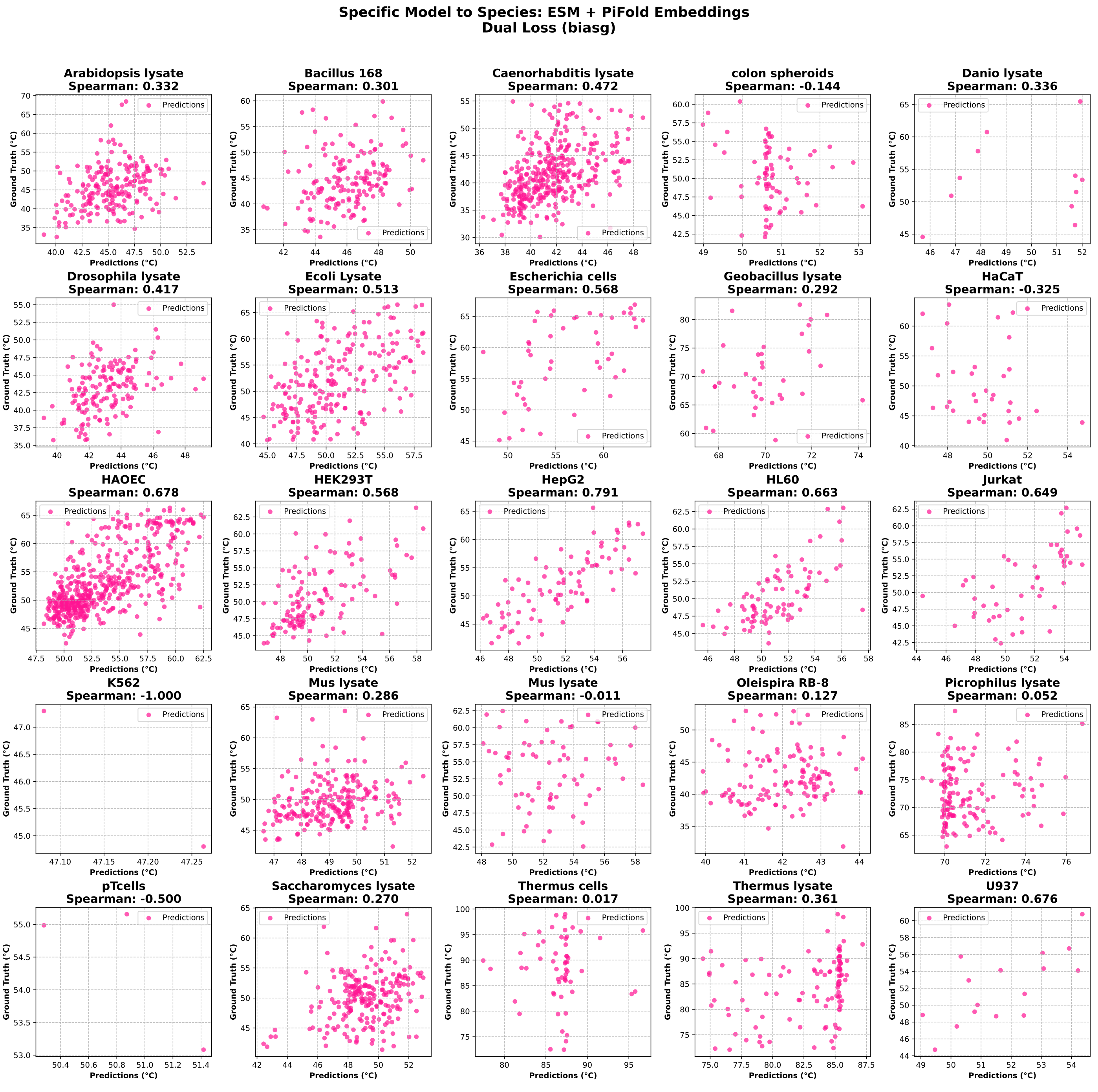

Figure S12: Scatterplot of the species-specific, dual-loss setting: using the dual loss function in combination with a concatenation of ESM and PiFold embeddings

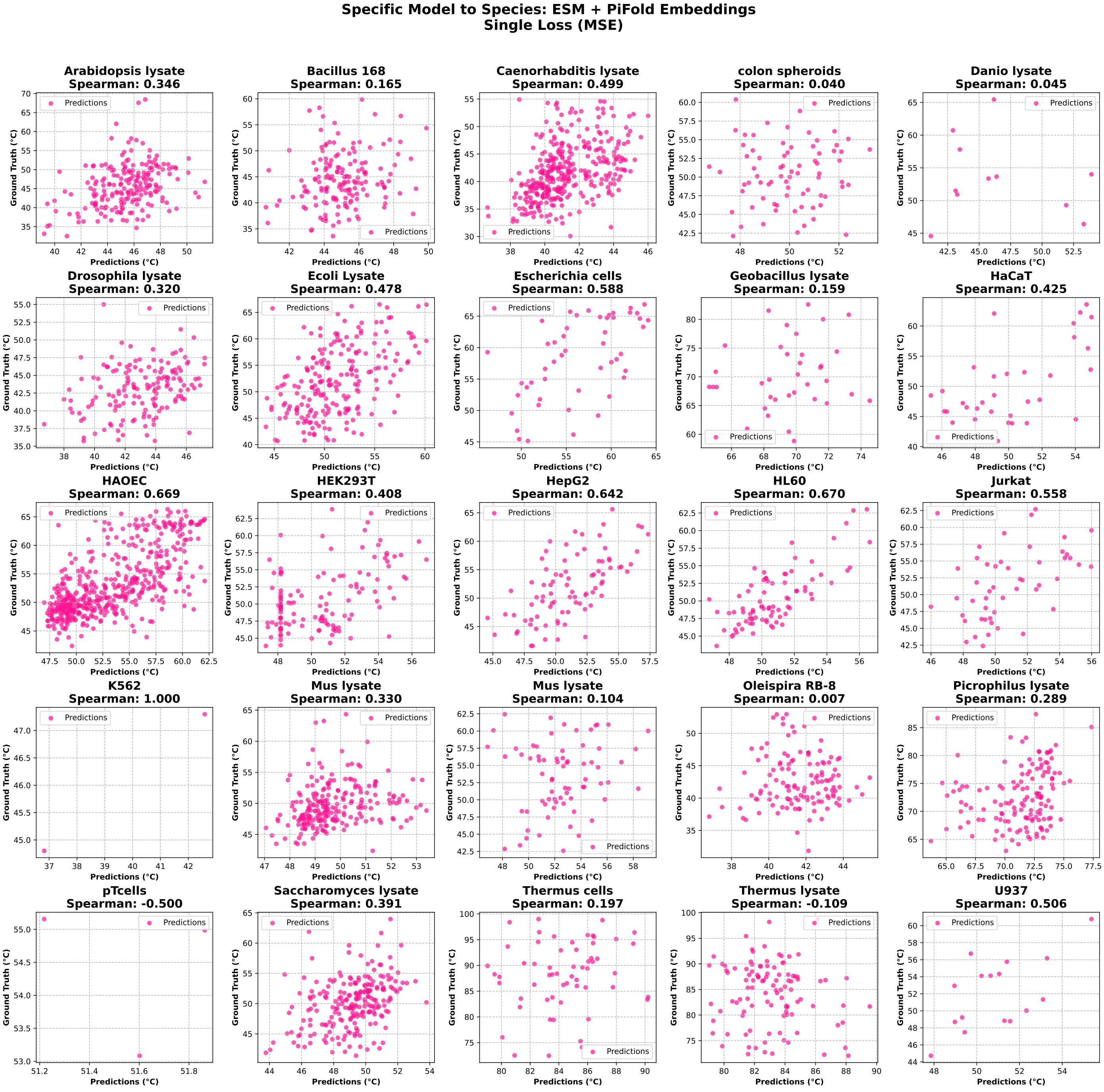

Figure S13: Scatterplot of the species-specific, MSE setting: a standard mean-squared error loss combined with a concatenation of ESM and PiFold embeddings

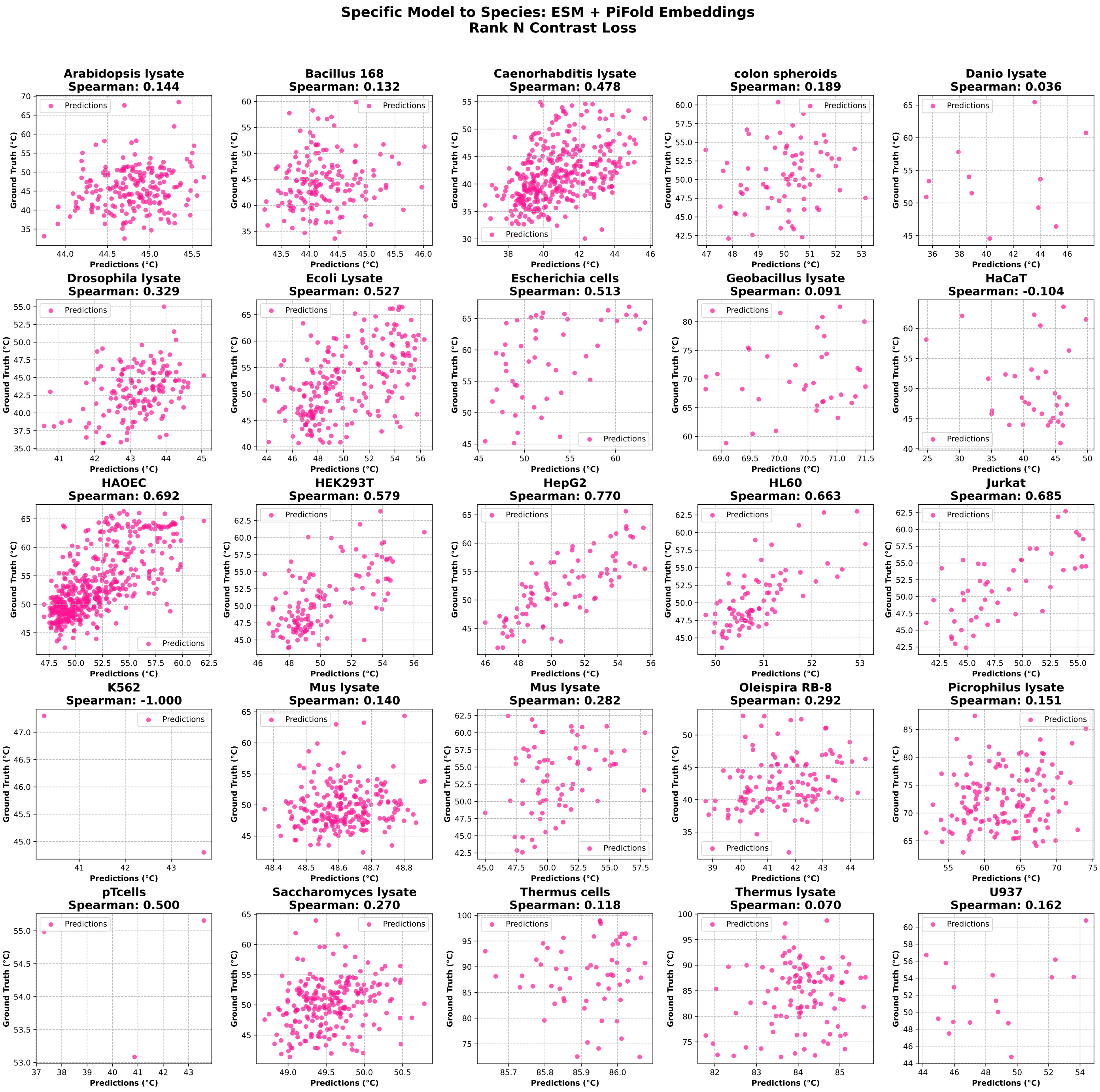

Figure S14: Scatterplot of the species-specific, rank-N-contrast setting: a contrastive representation learning approach combined with a concatenation of ESM and PiFold embeddings
